# The origins of species richness in the Hymenoptera: insights from a family-level supertree

**DOI:** 10.1186/1471-2148-10-109

**Published:** 2010-04-27

**Authors:** Robert B Davis, Sandra L Baldauf, Peter J Mayhew

**Affiliations:** 1 Department of Biology, University of York, York, YO10 5YW, UK; 2 Current address: Department of Zoology, Institute of Ecology and Earth Sciences, University of Tartu, Vanemuise 46, 51014 Tartu, Estonia; 3 Current address: Department of Evolutionary Biology, Uppsala University, Uppsala, Sweden

## Abstract

**Background:**

The order Hymenoptera (bees, ants, wasps, sawflies) contains about eight percent of all described species, but no analytical studies have addressed the origins of this richness at family-level or above. To investigate which major subtaxa experienced significant shifts in diversification, we assembled a family-level phylogeny of the Hymenoptera using supertree methods. We used sister-group species-richness comparisons to infer the phylogenetic position of shifts in diversification.

**Results:**

The supertrees most supported by the underlying input trees are produced using matrix representation with compatibility (MRC) (from an all-in and a compartmentalised analysis). Whilst relationships at the tips of the tree tend to be well supported, those along the backbone of the tree (e.g. between Parasitica superfamilies) are generally not. Ten significant shifts in diversification (six positive and four negative) are found common to both MRC supertrees. The Apocrita (wasps, ants, bees) experienced a positive shift at their origin accounting for approximately 4,000 species. Within Apocrita other positive shifts include the Vespoidea (vespoid wasps/ants containing 24,000 spp.), Anthophila + Sphecidae (bees/thread-waisted wasps; 22,000 spp.), Bethylidae + Chrysididae (bethylid/cuckoo wasps; 5,200 spp.), Dryinidae (dryinid wasps; 1,100 spp.), and Proctotrupidae (proctotrupid wasps; 310 spp.). Four relatively species-poor families (Stenotritidae, Anaxyelidae, Blasticotomidae, Xyelidae) have undergone negative shifts. There are some two-way shifts in diversification where sister taxa have undergone shifts in opposite directions.

**Conclusions:**

Our results suggest that numerous phylogenetically distinctive radiations contribute to the richness of large clades. They also suggest that evolutionary events restricting the subsequent richness of large clades are common. Problematic phylogenetic issues in the Hymenoptera are identified, relating especially to superfamily validity (e.g. "Proctotrupoidea", "Mymarommatoidea"), and deeper apocritan relationships. Our results should stimulate new functional studies on the causes of the diversification shifts we have identified. Possible drivers highlighted for specific adaptive radiations include key anatomical innovations, the exploitation of rich host groups, and associations with angiosperms. Low richness may have evolved as a result of geographical isolation, specialised ecological niches, and habitat loss or competition.

## Background

One of the greatest challenges in evolutionary biology is to explain heterogeneity in species richness amongst taxa, and in particular why a few notable taxa comprise the majority of species [1-4]. With over half of all described species, the insects pose perhaps the most obvious target group for biologists attempting to tackle this problem [5]. In this paper we address the phylogenetic location of shifts in diversification within one of the largest insect orders, the Hymenoptera (bees, ants, wasps and sawflies), containing some eight percent of all described species.

Phylogenies are useful tools for understanding the evolution of species richness. Since they specify shared common ancestry and absolute or relative taxon age they allow appropriate comparisons to be made amongst taxa, [6-8]. Taxon age in turn is important because for a given positive net rate of cladogenesis, species richness will increase over time. Thus, the species richness of a taxon can only be identified as anomalous if its absolute or relative age is also known. The cladistic and molecular revolutions, which have advanced phylogenetic information, have also therefore stimulated the development of statistical techniques that can best use the available phylogenetic information for macroevolutionary inference [1-5].

One of the most useful pieces of macroevolutionary information that can be extracted from a phylogeny is the identity of clades that are different, relative to others, in their rates of speciation and/or extinction. Once the identity of these exceptional clades is known, hypotheses about underlying causes may be tested [9], for example relating to adaptive radiations [10] or key innovations [11] although this may not always be straightforward [12]. Within the insects, some studies have attempted to do this at level of order or family [13-15], but within orders macroevolutionary studies have generally focussed on a small subset of taxa [16-20], which places obvious constraints on the explanatory potential of the study. A notable exception is the study of Hunt et al. [21] using a phylogeny of nearly 2,000 beetle species to estimate shifts in diversification across the order. Consistent with a comparable study across the angiosperm families [22], they detected numerous, both positive and negative, shifts in diversification.

There are four insect orders with over 100,000 described species, of which the Hymenoptera is one [23-25]. Little work has addressed the evolutionary origins of this diversity. Below family-level, ant diversification has been attributed to co-evolution with angiosperms [19], fig wasp diversification has been attributed to co-speciation with their host plants [26], nematine sawfly diversification has been linked to the exploitation of a larger host plant range [27], and the limited diversification of bumble bees has been attributed to biogeographical constraints [18], but the influence of such factors at higher taxonomic levels within the order have not been investigated. Studies addressing the role of parasitism in insect diversification more generally have also included hymenopteran data. For example, Mitter et al. [28] show increased diversification in phytophagous groups of insect (including the hymenopterous sawflies), but the same authors [29] found no evidence to suggest carnivorous parasitism (including the hymenopterous Parasitica) itself, enhanced diversification.

Clearly, if studies of Hymenoptera macroevolution are to be conducted at large taxonomic scales, appropriate phylogenies will be needed. Few large-scale hymenopteran phylogenies have been produced, and those that have are based solely on morphological data [30-34]. The most recent large-scale morphological phylogeny was constructed in 1999 [34]. Since 1999, and Ronquist's influential "State of the Art" hymenopteran phylogenetics symposium [35] a wealth of Hymenoptera phylogenies have been published. Perhaps most significant is the increased contribution of molecular work, in its infancy in 1999, alongside morphological work. Most molecular work however, has been fairly focussed, concentrating on certain subsets of hymenopteran families from the two traditional suborders Symphyta (sawflies) [36,37], or Apocrita (bees, ants and wasps) [38,39], or at even lower taxonomic levels such as superfamily [40,41].

**Figure 1:**
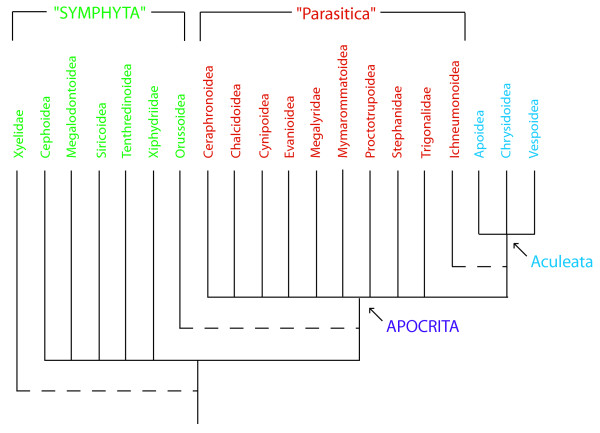
**A stylised summary of hymenopteran relationships**. Traditional suborders represented in capital letters. Terminal taxa indicate superfamilies, or those families not assigned to a superfamily. Dashed lines indicate hypothesised sister group relationships.

Symphyta are now accepted as a paraphyletic grade of taxa within which Apocrita is nested. Within this symphytan grade, the family Xyelidae are well supported as the sister group to all other Hymenoptera, and Orussidae are often supported as the sister lineage to Apocrita [34,42,43]. Within Apocrita there is a further subdivision into the Parasitica (parasitic wasps) and Aculeata (bees, ants and stinging wasps). Again, Parasitica is accepted now as paraphyletic, with Aculeata nested within it [24,34,44] (Figure 1). The Ichneumonoidea (Ichneumonidae + Braconidae) is often considered to be the sister group to Aculeata [24,44]. Apocritan relationships are however controversial and finding a consensus on familial and even superfamilial relationships is difficult [44]. For example, the validity of some superfamilies (e.g. Proctotrupoidea) is questionable [24,44] and relationships within other more accepted superfamilies (e.g. Chalcidoidea) are highly debateable [24,40,45,46].

Here we take available molecular, morphological and palaeontological information to construct a family-level supertree of the Hymenoptera including extinct taxa, presenting, in terms of the scope of its evidence base, the largest phylogenetic analysis of Hymenoptera to date. The supertree is used to examine the points at which Hymenoptera have experienced significant shifts in diversification. We then explore possible reasons for these shifts in the light of previous hypotheses, and their ability to explain Hymenoptera species richness.

## Methods

### Taxonomy

For consistency with other analyses by us on other orders [13] the taxonomic nomenclature is taken from a family list generated from Gordh & Headrick [47] for living families. There are slight differences between this list and other authoritative lists for Hymenoptera (see additional file 1): the list generally follows Gauld & Bolton [48], although departs significantly in splitting Apidae (*sensu lato*) into several families, following Goulet & Huber [49]. Gordh & Headrick [47] also include taxa that were recognised prior to Gauld & Bolton [48] but have since been assigned subfamily status by most researchers (e.g. Loboscelidiidae). By including such taxa in our input tree search, we allowed maximal opportunity for data coverage where phylogenetic studies distinguish them. Where possible we also included extinct families (despite their exclusion from diversification analyses below). A benefit of including extinct taxa is that they can help resolve phylogenetic problems [50], and may aid future attempts to date the tree. We used the nomenclature of Ross & Jarzembowski [51] and the regularly updated EDNA Fossil Insect Database http://edna.palass-hosting.org/search.php for extinct families. Our diversification analyses were only conducted with extant taxa (see below). For a full list of taxa and alternative taxonomies see additional file 1.

### Input Trees

Input trees were sought online via Web of Science and Google Scholar using the following keywords: hymenoptera*, apocrita*, aculeata*, parasitica*, symphyta*, phylogen*, cladistic, cladogram. (* where any word begins with those letters). Further source studies were collected from the reference sections of these papers. Potential input trees from the collected literature were extracted manually and compiled into a dataset of hymenopteran phylogenies. Hennig [52] is often cited as a milestone in hexapod phylogenetics (synthesizing his initial cladistic views), and only studies post-1969 were considered. New papers on hymenopteran phylogeny are regularly published, so a cut-off date of December 2008 was used to facilitate future updates of our work.

A total of 101 input trees were obtained from the literature, which contain information on hymenopteran family relationships. However, not all of these could be retained for the final analysis as there is data non-independence (i.e. where different studies overlap in the primary data they use) between them. This is a major challenge with supertree analyses when they are used to summarise published phylogenies (but not when supertrees are used as phylogenomic tools [53]) as it is often impossible to fully remove data non-independence without unnecessary loss of other information. Following, and slightly modifying, the guidelines of Bininda-Emonds et al. [54] we attempt to minimise data non-independence as far as possible. Choosing which input trees to keep is done as follows:

• Where data overlap is under 50% we retain both trees

• Where data overlap is over 50% but taxon overlap is under 50% we keep both trees

• Where data and taxon overlap is 50% or over, we first take the author's preferred hypothesis, because supertrees are designed to synthesise current hypotheses.

• Where no tree is explicitly preferred, we take the most comprehensive tree, because supertrees are designed to be comprehensive.

• Where both trees are equally comprehensive we take the most recent tree, under the notion that inferences converge on the truth with time.

• Where both trees are equally recent, we use a tree that has support measures provided over one that does not, as a more reliable phylogeny.

• Where trees cannot be chosen based on the above criteria we use all available trees and downweight them in the final analysis accordingly.

Of the 101 trees in the dataset, 24 were removed based on the above protocol. Of the families listed in Gauld & Bolton [48], only four were absent from input trees: the Rotoitidae (Chalcidoidea), the Eucoilidae (Cynipoidea), the Peredeniidae (Proctotrupoidea) and the Austroniidae (Proctotrupoidea). For a list of input trees and data non-independence see additional file 2. Input tree files are available on request from RBD.

Within these trees, valid taxa (see taxonomic nomenclature) were sometimes portrayed either as paraphyletic or polyphyletic, for example in molecular data sets where a single family could be represented by several species. In these instances polyphyletic families were removed entirely as their placement is uncertain. Paraphyletic families were condensed into a single branch. This issue could be dealt with in other ways. For example weighted sets of input trees representing alternative placements of such taxa could be used. Our approach is more pragmatic than this. Alternatively, such taxa could be entirely removed from supertree analysis, but this could result in unnecessary loss of information. The effects of other approaches to dealing with this issue are unknown.

### Supertree Methods

Many previous supertree studies [22,55-64] have used the popular matrix representation with parsimony (MRP) method [65,66] only, though some more recent studies use multiple methods [67,68]. We implement three supertree methods; two matrix-based and one distance-based, to avoid over-reliance on a single method, and to ensure we obtain as optimal a phylogeny as possible. In addition to MRP, we use matrix representation with compatibility (MRC (also known as split-fit)) [69], and the average consensus method [70]. Although the average consensus approach can use branch length information, here we do not as many trees do not have such information available. We avoided using strict supertree methods as they only identify relationships common to all input trees [71]. For software and settings see additional file 3.

Analyses of the complete data were successfully conducted using the average consensus and MRC methods. However, analysis of the full data set could not be conducted using MRP. There is a high amount of conflict in the dataset, which makes the analysis more computationally challenging. Furthermore poorly represented taxa (i.e. with much missing data in the MRP matrix) cloud the analysis. We note that different supertree methods are implemented in different software and the number of optimal trees returned will reflect not only the data but also the efficiency of tree searching. To enable analysis using MRP, in addition to MRC and the average consensus the dataset was split into three widely accepted subgroups within Hymenoptera: Symphyta, Parasitica and Aculeata. Since Symphyta and Parasitica are likely not monophyletic (Figure 1) the most widely represented member of the relevant nested clade, here Vespidae, was included in these analyses to test for paraphyly and to provide a reference point in order to graft the three parts of the hymenopteran tree together. Including more nested aculeate families has no effect on the outcome of the analyses. Separate supertree analyses were run for these three groups of hymenopteran taxa. The following summarises the analyses undertaken using each technique:

• Standard MRP: Separate Symphyta, Parasitica and Aculeata supertrees produced and grafted together. This constrains the monophyly of Aculeata and Apocrita. Due to large amounts of conflict in the data set, Parasitica families that were underrepresented (i.e. < 10 pseudocharacters in the matrix) or only in input tree polytomies had to be removed to ensure the analysis could be completed. Fully bifurcating trees were required for our diversification analysis. Extended majority rule trees display all relationships in 50% or more of the most parsimonious trees (MPTs) recovered in supertree analysis. Where less than 50% of these MPTs agree on one relationship, the relationship with the largest proportion of sub-50% agreement between MPTs is displayed producing a fully bifurcating tree. In the extended majority rule setting symphytan and aculeate taxa in polytomies were additionally removed. Their inclusion cannot be justified as the extended majority rule method will arbitrarily place them in bifurcating splits without any precise information on this.

• MRC: Separate Symphyta, Parasitica and Aculeata supertrees were produced. Problematic Parasitica did not need removing, but could be removed for direct comparison with standard MRP results. Analyses were also run using the all-inclusive dataset and the supertree compared to the re-grafted one to test the effects of constraining monophyly. In the extended majority rule setting symphytan and aculeate taxa in polytomies were additionally removed.

• Average consensus: Separate Symphyta, Parasitica and Aculeata supertrees were produced. Underrepresented Parasitica did not need removing, but all taxa only in polytomies were removed as the average consensus method produces only one fully bifurcating tree. The reasons behind the removal of these taxa are the same as if an extended majority rule tree was to be constructed (see above). Analyses were also run using the all-inclusive dataset.

We use the V index of Wilkinson et al. [72] to measure support. This considers the number of input trees in agreement and in conflict with relationships in the supertree on a scale running from -1 (all conflicting) to +1 (all supporting). For a given relationship in a supertree, each input tree can either support or conflict with this, where the taxa involved in the relationship are represented. Each input tree can therefore contribute a score of +1 (indicating support) or -1 (indicating conflict) to the score for a relationship in a supertree. All +1s and -1s are added and a mean calculated to provide scores for individual relationships. The mean of these nodal scores can also be calculated to provide an overall V score for a supertree allowing comparison between supertrees produced using different methods. V+ is a more liberal score, which considers permitting relationships in input trees (e.g. polytomies) as support (+1), and is also used here. V scores were calculated using the software stsupport (obtained in January 2007 from http://webspace.qmul.ac.uk/jacotton/software/stsupport.html#Download). For clarity of presentation we use branch thickness to visualise support for relationships in figures and provide a table of V and V+ scores in additional file 4. An alternative supertree-specific support measure, rQS [73,74] was not used as this has been found to be unreliable [75,76]. Output files are available on request from RBD.

### Diversification Analysis

**Figure 2:**
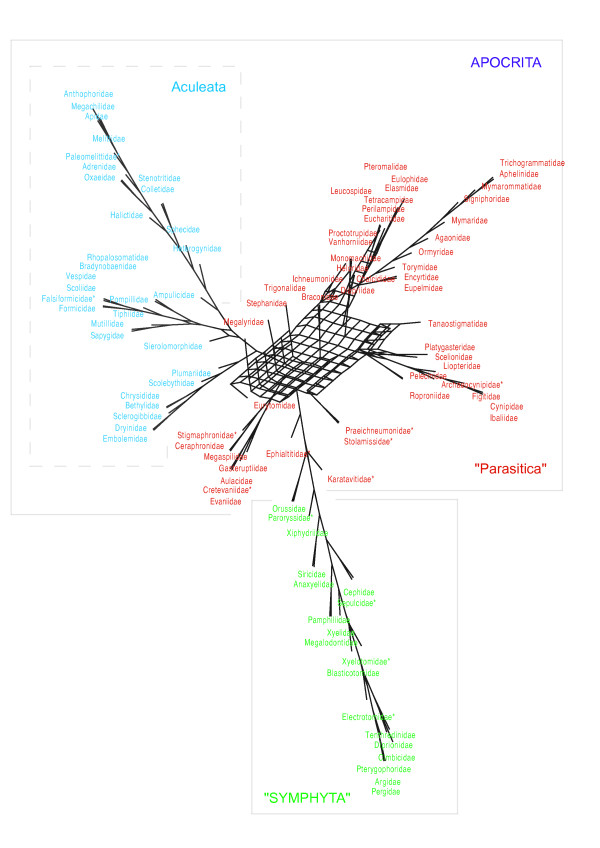
**A consensus supernetwork highlighting the uncertainty of phylogenetic relationships between hymenopteran families**.

Fully bifurcating extended majority rule trees were used for analysing shifts in diversification within the extant lineages of Hymenoptera. The MRP and MRC trees had similar positive V support (see additional file 5), but as we wished to use the most complete a tree for diversity analyses, we chose MRC supertrees over those produced by standard MRP. We were able to retain more taxa for MRC analyses and as we could run an all-inclusive analysis (without needing to constrain any clades), this is arguably a preferable approach. The average consensus tree was poorly supported (see additional file 5) and not used for diversification analyses. The two MRC trees differ greatly in deeper apocritan relationships (shown as a consensus network in Figure 2). Given that these relationships are weakly supported, both were used in diversification analysis to test the effects of alternative phylogenetic hypotheses on our findings, and therefore determining which shifts in diversification are robust to changes in the phylogeny.

We compared sister taxon species richness to assess where significant shifts in diversification have occurred during the evolution of the Hymenoptera, as used previously in studies of insect species richness [13,14]. This analysis does not require information on dates of branching events, which we have not attempted to infer. If sister taxa radiate at equal, but not necessarily constant, rates over time, including extinction [77] all possible sizes of splits of *N *species between these two clades are equally likely [78]. The two-tailed probability of an equal or greater magnitude of split under the null hypothesis of equal net diversification rates is given by 2 [*N*_*small*_/(*N*_*small *_+ *N*_*large *_- 1)].

**Figure 3:**
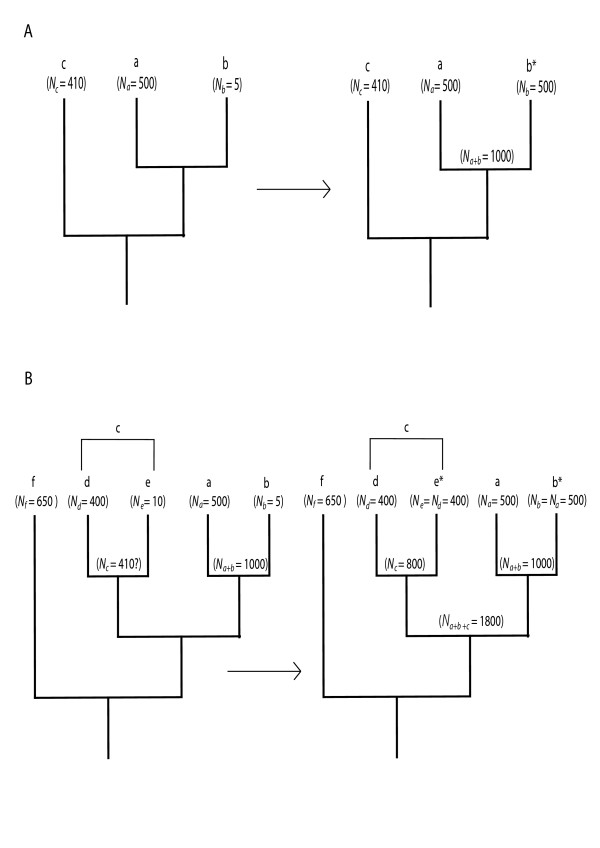
**The method of Davies et al. (2004) explained**. In Figure 3A taxa *a *and *b *have significantly different species richnesses (*N*). To detect the direction of the shift here we compare *N*_*a *_and *N*_*b *_to *N *of their nearest outgroup *c*. As *N *does not differ significantly between *a *and *c*, but does between *b *and *c*, we have detected a significant downshift in species richness associated with *b*. Figure 3B shows a more complicated scenario, where taxon *c *is made of two taxa *d *and *e*, which have significantly different species richnesses and they themselves need comparing to their outgroup (i.e. *a *+ *b*). However, it is not possible to compare the values for *N*_*a*_, *N*_*b*_, *N*_*d *_or *N*_*e *_as they are relative outgroups to one another in which we have not been able to detect the direction of the significant shift. In such circumstances, the combined *N *of species rich taxa (for example *a *and *d*) are compared to *N *of the next outgroup *f*. The same goes for the species poor taxa (for example *b *and *e*). In this example it is *N*_*b *_+ *N*_*e *_which is significantly different to *N*_*f *_and we have therefore detected significant downshifts in taxa *b *and *e*.

Care must be taken when interpreting the results of this analysis, as a seemingly significant difference in species richness at a basal node may be attributable to such an occurrence towards the crown (i.e. a "trickle-down" effect). We therefore use the method of Davies et al. [22] to account for this (see also Hunt et al. [21]), which works using a contingent species-richness correction algorithm (Figure 3). Such species-richness correction methods have been shown to be more valid than a range of alternatives in simulations [79], and produce intuitively sensible results [13,14,21,22].

Species richness for most families was taken from Goulet & Huber [49]. Most of the values are estimates of described species richness. However, the number of described species is not accurately known for the two largest families, Braconidae and Ichneumonidae. Estimates vary markedly from source to source, and both are known to be markedly underdescribed. We therefore also follow Goulet & Huber [49] in using the widely cited estimates of 60,000 spp. for Ichneumonidae [80] and 40,000 spp. for Braconidae [81], which although far larger than the number of described species are still possibly underestimates of true species richness. Alternative lower estimates have no effect on the results below. If estimates were not given in Goulet & Huber [49], they were taken from Parker [82], except for Stenotritidae [83] and Pterygophoridae [84]. Lastly, an outgroup to all Hymenoptera was required in order to test for a significant difference in species richness at the basal node of the tree. Whether we take the panorpidan orders (Diptera, Lepidoptera, Mecoptera, Siphonaptera, Strepsiptera and Trichoptera) [76,85,86] or all other Holometabola [87-89] as the sister group to Hymenoptera, has no effect on our overall findings. We take a combined described species estimate from Grimaldi & Engel [24] for this purpose.

## Results

**Figure 4:**
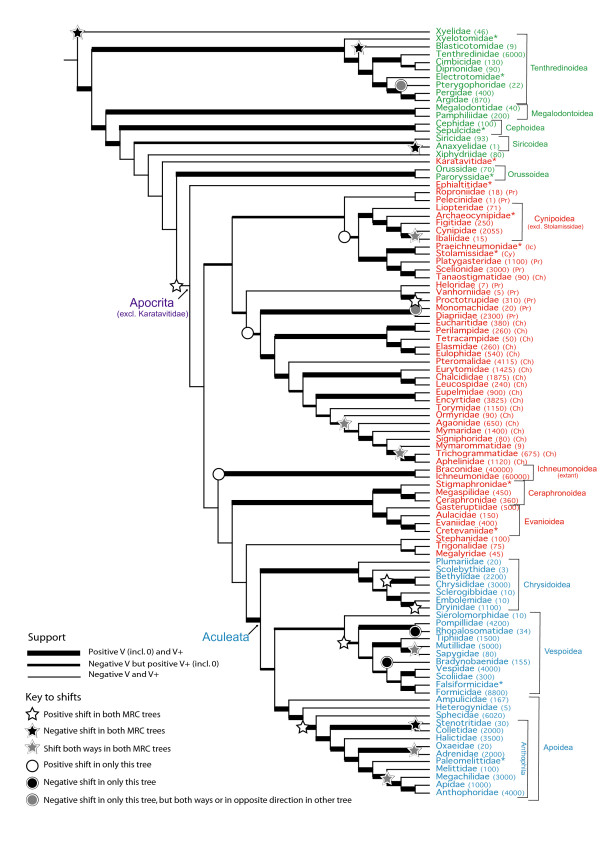
**The extended majority rule MRC supertree of hymenopteran families from an all-in analysis**. Numbers in brackets next to extant families indicate number of species. Membership of families with non-monophyletic "superfamilies" indicated as follows - Ch = Chalcidoidea, Cy = Cynipoidea, Ic = Ichneumonoidea, Pr = Proctotrupoidea. Taxa colour coded in relation to previous figures: Symphyta - green, Parasitica - red, Aculeata - blue.

Whilst relationships at the tips of the supertrees tend to be well supported, those along the tree backbone are often less so, notably amongst the Parasitica superfamilies (e.g. Figure 4). The paraphyly of Symphyta and Parasitica is supported, as is the monophyly of Aculeata. Apocrita appear monophyletic (ignoring the position of the poorly represented fossil family Karativitidae in one analysis), with Orussidae as their sister in all analyses. The monophyly of most superfamilies is supported, but Mymarommatidae (often afforded separate superfamily status) nest within Chalcidoidea. Our analysis also provides no support for the monophyly of Proctotrupoidea (Figure 4).

Ten significant shifts in diversification (six positive and four negative) are found common to both MRC supertrees (Table 1, Figure 4, additional file 5). The most inclusive shift is the suborder Apocrita. Apocrita contain over 180,000 species, compared to the 70 of Orussidae, although most of these species can be attributed to shifts in diversification further up the tree (Table 1).

**Table 1:** Shifts observed in supertrees from all-in and compartmentalised MRC analyses.

Group in which shift occurs. Species numbers in brackets (overall/those involved in shift (all-in analysis))	Direction of shift	Sister group. Species number in brackets (overall/those involved in shift (all-in analysis except *))
Xyelidae (46/46)	-	Other Hymenoptera (190,735/18,266)
Blasticotomidae (9/9)	-	Other Tenthredinoidea (with or without Xyelotomidae) (7,512/8,760)
Anaxyelidae (1/1)	-	Siricidae (93/93)
Apocrita (182,630/4,792)	+	Orussidae (70/70)
Proctotrupidae (310/310)	+	Vanhorniidae (5/5)
Bethylidae + Chrysididae (5,200/5,200)	+	Sclerogibbidae + Dryinidae + Embolemidae (1,120/30)
Drynidae (1,100/1,100)	+	Embolemidae (10/10)
Vespoidea (excl. Sierolomorphidae) (24,069/41,180)	+	Apoidea* (21,842/177) or Sierolomorphidae (10/10)
Anthophila + Sphecidae (21,670/23,640)	+	Heterogynaidae (5/5)
Stenotritidae (30/30)	-	Colletidae (2,000/2,000)

Two-Way Shifts (Both Trees)		

Ibaliidae (15/15)	-/+	Cynipidae (2,055/2,055)
Ormyridae (90/90)	-/+	Agaonidae + Mymaridae + Signiphoridae + Mymarommatidae + Aphelinidae + Trichogrammatidae (3,934/3,934)
Mymarommatidae (9/9)	-/+	Aphelinidae + Trichogrammatidae (1,795/1,795)
Sapygidae (80/80)	-/+	Mutillidae (5,000/5,000)
Oxaeidae (20/20)	-/+	Adrenidae (2,000/2,000)
Melittidae (100/100)	-/+	Anthophoridae + Apidae + Megachilidae (8,000/8,000)

Note: Sometimes the number of species apparently involved in a shift is greater than the observed number for a clade (e.g. Vespoidea), where subsequent negative shifts have prevented species richness getting as high as projected. Species richness calculations can be found in additional file 9.

The Vespoidea (excluding Sierolomorphidae) and the Anthophila + Sphecidae (forming the majority of Apoidea) are also recovered as significant positive shifts in diversification with possibly sizeable effects on species richness (Table 1). Other shifts are confined to smaller clades comprising only one or two families: upshifts in diversification in Bethylidae + Chrysididae, in Dryinidae, and in Proctotrupidae, and downshifts in diversification in Stenotritidae, Anaxyelidae, Blasticotomidae and Xyelidae. Only one of these shifts is linked to a phylogenetic topology not recovered in the standard MRP tree: in the MRP tree Sierolomorphidae nests within Vespoidea as sister to Formicidae + Falsiformicidae (i.e. ants), and not at the base of this superfamily.

In addition to these positive and negative shifts in diversification are six examples of a significant difference in species richness between sister taxa, but not a significant difference with respect to their outgroup. Two of these involve Chalcidoidea families, two involve Apoidea families, and one each involves cynipoid and vespoid families (Figure 4). Each tree also produces a small number of shifts in diversification not found in the other tree (see additional file 6); these are generally away from the backbone of the tree with the exception of a possible positive shift of all Hymenoptera to the exclusion of Xyelidae in the compartmentalised MRC tree. Many of these are poorly supported groups or the relationship with their sister group is poorly supported (see V scores in additional file 4).

## Discussion

We have produced a family-level phylogeny of Hymenoptera that is the most comprehensive to date in terms of its evidence base. We include almost all living and many fossil families. As a result we have also identified several likely shifts in diversification. Below we first discuss the implications of our trees for the future of Hymenoptera phylogenetics. We then discuss the implications of the identified shifts in diversification.

### Hymenopteran Phylogeny

This supertree approach has yielded findings both supporting and questioning previous hypotheses across the hymenopteran tree. Several relationships receive full support (V = 1) and appear in both MRC trees, indicating that the phylogenetic evidence, as used in our analysis, is in full agreement: Argidae + Pergidae, Cephoidea, Orussoidea (Symphyta); Aphelinidae + Trichogrammatidae, Ceraphronoidea, Megaspilidae + Ceraphronidae, Evaniidae + Cretevaniidae (Parasitica); Anthophila, Megachilidae + Apidae + Anthophoridae (Aculeata). In addition to these, two sets of relationships receive a V score of over 0.8: Argidae + Pergidae + Pterygophoridae (Symphyta), and Eupelmidae + Encyrtidae (Parasitica). Furthermore, symphytan superfamily relationships are consistently recovered with a mostly well-supported branching order in supertree analyses of (Xyelidae, (Tenthredinoidea, (Megalodontoidea, (Cephoidea, (Siricoidea, (Xiphydriidae, (Orussoidea + Apocrita))))))), with Xiphydriidae outside of any existing superfamily. The latter has often remained unclassified at a superfamily level [49]. Here we find no evidence to place it in an existing superfamily either, and its position, is in agreement with more recent symphytan studies [37,42] Full V scores are provided in additional file 4. Hence, our analyses suggest that the above sets of relationships can be regarded as well known and supported.

The most concerning lack of resolution regards apocritan superfamily relationships, and is highlighted by two MRC and one MRP trees with alternative topologies (Figures 2, 4, additional files 7, 8). However, the shifts in diversification we identified earlier are stable to these differences (see below). No group is strongly supported as the sister group to Ichneumonoidea. Tentatively, the Stephanidae might be closely related as in both MRC and MRP analyses they are either singularly or in a more inclusive clade as sister to Aculeata yet this is poorly supported (V = -0.728 or -0.642 respectively). Furthermore, the validity of some traditionally recognised superfamilies is uncertain. Not all extended majority rule analyses show Vespoidea to be monophyletic. Vespoid monophyly has previously been questioned [48], though based on features of Pompilidae (here shown to group in Vespoidea) and not Sierolomorphidae, which may sit outside Vespoidea.

Arguably our most interesting finding lies in the phylogeny of Chalcidoidea and Proctotrupoidea. Chalcidoidea are usually well accepted as a superfamily [24,90] but here Mymarommatidae (the single family in Mymarommatoidea) always nest within them with good support in agreement with Krogmann & Vilhelmsen [46]. The status of Mymarommatoidea is dubious though. It has previously been included within Chalcidoidea when Mymarommatidae nests within them, and as a separate superfamily otherwise. Its taxonomy seems phylogeny-dependent, and results here suggest Mymarommatidae be included in the Chalcidoidea [47]. Despite this, the Chalcidoidea are not always recovered as monophyletic (position of Tanaostigmatidae - Figure 4), but evidence in input trees for this is lacking (V = 0).

The validity of Proctotrupoidea has previously been questioned, for example in some input studies [39,41] and through agreement in supertree analyses there may be a case for revising their higher taxonomy. It is of interest to see which families consistently group together as a basis for revising the taxonomy of this group, though we do not here suggest any specific revisions. With strong support (V = 0.6) Proctotrupidae and Vanhorniidae are recovered as sister groups in agreement with Castro & Dowton [91] and Dowton & Austin [39] with Heloridae basal to this in all analyses. Diapriidae and Monomachidae are recovered in all analyses with reasonable support (V = 0.179), while Roproniidae and Pelecinidae are recovered in all analyses but with poor support (V = -0.234). This relationship is also in agreement with Castro & Dowton [91] and Dowton & Austin [39]. These problematic relationships and arguably a revision of "proctotrupoid" taxonomy should be priorities for future research.

### Diversification Shift Analyses

Because we have conducted the first quantitative tests of shifts in diversification at higher taxonomic levels in Hymenoptera, most of the inferred shifts are entirely novel hypotheses. Two important resulting questions are: how robust are these inferences, and what might have caused them? Whilst our analyses can directly inform us about the former, the phylogenetic positions of shifts constrain their possible causes but are not themselves hard evidence about causes: any difference between the two clades concerned might be responsible. A particular problem is that some ecological differences between clades noted below, such as the taxonomic extent of hosts and geographical distribution, might have caused shifts in diversification or might equally well be the result of them. Nonetheless, such differences help us to raise hypotheses for further tests.

The deepest consistent shift in diversification we infer, although not having the greatest effect on species richness (Table 1; for species richness calculations see additional file 9), occurs at the origin of Apocrita. The sister pairing of Orussidae (plus Paroryssidae) and Apocrita receives poor support like most of the nodes along the supertree backbone, but this relationship does appear in several studies where it is often strongly supported [33,37,42,43,92]. Where alternative sister-pairings exist based on both morphological [30] and molecular data [36,93] the Apocrita are still shown to be sister to another single symphytan family and our findings are not affected.

Orussidae are the only carnivorous parasitic symphytans [83]. Apocritan taxa are united by the move to a more refined carnivorous parasitic lifestyle, which probably originated out of an orussid-style life cycle. Therefore it is possible that carnivorous parasitism is a prerequisite for other key innovations, which are discussed below (i.e. a case of contingent radiation [12]). Apocritan larvae have a closed hindgut [44,94,95]. This might function to increase assimilation of nutrients or avoid fouling the host [95], and avoid detection by ovipositing orussids, which use faeces to detect hosts [96]. In the adult, the constriction between abdominal segments 1 and 2, (i.e. the "wasp waist") is a defining feature of apocritan taxa [44,49,94]. This might function to facilitate oviposition or improve flight [48]. Furthermore, Sharkey [44] suggests that the variety of ovipositor morphologies has had a key role in facilitating the diversity of apocritan species and may represent an adaptive radiation towards using a range of host species.

We observe a positive shift in diversification the Vespoidea (Table 1) to the exclusion of Sierolomorphidae in both MRC trees despite different proposed sister groups (either Sierolomorphidae (10 species) or Apoidea). The shift detected could have led to the origin of over 40,000 species had negative shifts in diversification further up the tree not occurred (where these are detected depends on the phylogeny used). Though a sister relationship with Apoidea gains greater support, the size of the potential shift in diversification is greater when Sierolomorphidae are the sister group (p = 0.0004 in contrast to 0.0146). The other Vespoidea differ from Sierolomorphidae in a few anatomical features, though they lack the constriction observed in other Vespoidea (except Pompilidae) between the second and third abdominal segments [48,49]. This extra vespoid constriction may act to enhance any dexterity conferred by the apocritan "wasp waist" in an example of contingent radiation [12], and could enable effective use of the sting in defence [97]. The specialised articulation between the second and third abdominal segments is also absent from Apoidea.

The Anthophila (bees) and Sphecidae, together containing over 21,500 species, form a well-supported clade (V = 0.467) and together represent a positive shift in diversification. The taxonomy used here [47] includes sphecid subfamilies which have family status elsewhere (e.g. Crabronidae, Pemphredonidae, Astatidae). Even when the species numbers, which these families contribute to our Sphecidae, are removed (a ten-fold reduction), this positive shift is still observed. In a similar, but less drastic, manner to that observed in Vespoidea, the potential number of species we might have expected from this shift could be greater (another potential 2,000 species) had negative shifts in diversification further up the tree not occurred. Their sister group Heterogynaidae (well supported, V = 0.467) constitutes just five species and is poorly known. While heterogynaid females are brachypterous (i.e. have reduced, non-functional wings) [49,98] those of the Sphecidae and the bees are not [48,49]. Reduced wings may result in reduced dispersal. Heterogynaidae species are found only from Madagascar to the Mediterranean [98], whereas bees and sphecids have a wider distribution [49]. Furthermore, adult sphecids feed at flowers as do all bees, but whilst larvae of bees are also phytophagous, the larvae of sphecids retain a carnivorous habit [99]. A move towards an association with angiosperms has been cited as a reason for insect diversification in similar studies on other groups [17,19,20].

The Bethylidae and Chrysididae are a fairly well-supported sister pairing (V = 0.111) and combined have experienced a significant positive shift in diversification compared to their sister group of Sclerogibbidae, Dryinidae and Embolemidae (Table 1). The monophyly of these five families is not well-supported (V = -0.091, but V+ = 0.333), but these taxa are sometimes in polytomies [32,34], and Bethylidae and Chrysididae appear in trees that do not include the other families and are consequently shown to be sister to alternative families [100,101]. Bethylids are parasitoids of Lepidoptera and Coleoptera, while chrysidids are parasitoids primarily of other Hymenoptera and Lepidoptera. These host groups are themselves extremely speciose, compared to the host groups of Sclerogibbidae (Embioptera hosts), Dryinidae and Embolemidae (both Homoptera hosts) [48,49].

A significant positive shift is observed in the Dryinidae compared to their sister family Embolemidae (Table 1), a sister pairing that receives strong support (V = 0.667). Most dryinids possess an enlarged claw on the female protarsus, which could confer increase raptorial ability, consequently enhancing capacity for parasitisation [49,102]. The host groups of Dryinidae are two large superfamilies of Hemiptera (Cicadelloidea and Fulgoroidea) [48], making their potential ecological niche broad.

The Proctotrupidae (310 species) are a positive radiation relative to their sister group Vanhorniidae (5 species), a sister pairing with good support. The Vanhorniidae are parasites on just one particular coleopteran family - Eucnemidae (1100 species [47]), while the Proctotrupidae parasitise members of the Diptera and Coleoptera [48] including two of the largest coleopteran families Carabidae (20,000 species) and Staphylinidae (32,000 species - [47]).

The only significant negative shift occurring within the Apocrita concerns the family Stenotritidae (Table 1). This family is sister to Colletidae (V = 0.5). While Colletidae (comprising 2,000 species) has a wide distribution through the southern hemisphere (with genera *Colletes *and *Hylaeus *wider still [103]), Stenotritidae is found solely in Australia [49].

Three symphytan families have likely undergone significant negative shifts in diversification. The Anaxyelidae comprises just one modern species (*Syntexis libocedrii*) surviving from a previously larger Mesozoic/Cenozoic group [104]. It is sister to Siricidae (V = -0.270 but V+ = 0.316). *Syntexis libocedrii *has a very specialised lifestyle, where the female oviposits only into recently burnt or still burning cedar wood specifically [105]. The Blasticotomidae, comprising 9 species, also occupies a specialist niche. Blasticotomidae larvae exhibit a phloem-feeding strategy known as "phloemining", which is unique amongst modern families. This habit may have been more widespread previously within tenthredinoid families [106]. They are strongly supported as sister to the other Tenthredinoidea (either including or excluding the extinct family Xyelotomidae), which comprises over 7,500 species (V = 0.556 or 0.569 respectively).

The Xyelidae may also represent a significant negative shift in diversification away from the other Hymenoptera (over 190,000 species). How large this shift is inferred to be depends on the phylogeny studied, and therefore the shifts in diversification detected and adjusted for further up the tree. They are a relict family containing now just 46 species dating back to the Triassic. Carpenter [107] reports 36 fossil genera compared to the current five. Other relict groups are often inferred to have lost original niche space over time [108] or to have been hindered in further diversification by the radiation of more derived taxa [109]. In the compartmentalised MRC analysis, a positive shift is also detected in the remaining Hymenoptera. If this inference is true, it could be in part due to competitive replacement of Xyelidae. It is notable that there are no positive shifts in diversification detected amongst symphytan taxa, in contrast to the Apocrita.

In addition to the above shifts in diversification, which are all in one direction, there are six two-way shifts identified common to both MRC trees, where both sister taxa have shifted in opposite directions leading to a significant difference in species richness (Table 1). All occur at tipward nodes within Apocrita. Only one relationship of these six is dubious (Ormyridae v Agaonidae + Mymaridae + Signiphoridae + Mymarommatidae + Aphelinidae + Trichogrammatidae (V = -0.667)). Within this group, there is a two-way shift between Mymarommatidae and Trichogrammatidae + Aphelinidae. Mymarommatidae are globally distributed, but little is known of their biology [49]. Trichogrammatidae and Aphelinidae are egg parasites of taxonomically diverse insect groups including Lepidoptera and some Hemiptera. Similarly, Mutillidae use speciose host groups, while their sister group Sapygidae have a specialist cleptoparasitic lifestyle. Another positive shift is that of the gall-forming Cynipidae, compared to the negative shift in their sister group Ibaliidae, which are parasites of the species-poor Siricoidea [49]. The other two two-way shifts in diversification both occur in Apoidea. A negative shift in Oxaeidae, a geographically restricted group [110] occurs opposite a positive shift in Adrenidae, which have a wider distribution. Lastly, a negative shift in Melittidae occurs against a positive shift in Anthophoridae + Apidae + Megachilidae. Melittidae are oligolectic, feeding on a narrow range of host plants [111], and the other families comprise the long-tongued bees, which may represent an adaptive radiation to feed on flowers with more complex structures [112].

Some further shifts in diversification were inferred in the all-in analysis (Figure 4) but not the compartmentalized one, hence are more tentative. The Ichneumonoidea make up 100,000 species in a possible positive shift. This superfamily displays a number of innovations relating to parasitism (e.g. ovipositor steering mechanisms, polydnaviruses etc [113]). In the compartmentalised tree the Ichneumonoidea fall within a larger clade representing a positive shift. Other positive shifts in diversification are in large, but poorly supported groups with no obvious connection. Negative shifts in diversification may have occurred in Pterygophoridae and Monomachidae, which have restricted geography [49,114], and also in the poorly known Rhopalosomatidae and Bradynobaenidae.

## Conclusions

This study provides the most comprehensive phylogenetic framework of Hymenoptera to date with which to test evolutionary hypotheses. Such large-scale phylogenies must be regarded as work in progress since there are inevitably many topological uncertainties. With the current evidence we identify many shifts in diversification robustly, which have shaped the evolution of the group, from suborder, through superfamily, to family-level clades. The location of other shifts in diversification is hard to place given current phylogenetic uncertainty. Our findings support other large phylogenetic studies suggesting that the origin of species richness in large clades (such as beetles and angiosperms, and insects as a whole) are due to multiple smaller adaptive radiations with possibly diverse independent causes [13-15,21]. Thus, there has been much heterogeneity in net rate of change in species richness within Hymenoptera, without which their richness would certainly have been considerably lower, but also might in places have been higher. Notable differences between the clades involved often include morphological innovations in one of the groups, but also differences in host groups and geographic distribution, and these suggest causal hypotheses for future microevolutionary studies on speciation and extinction processes. Our tree provides a benchmark against which future large scale hymenopteran phylogenies can be evaluated.

## Authors' contributions

All authors have read and approved the final manuscript. RBD conducted supertree and diversification analyses and drafted the manuscript. PJM and SLB designed, obtained funds for, and participated in coordinating the study, and PJM helped to draft the manuscript.

## Supplementary Material

Additional file 1 **List of valid hymenopteran families**. A list of all families recognised as valid for supertree analysis, information on whether they could be included in the analysis or not, and synonyms also provided. Click here for file

Additional file 2 **Input trees and data non-independence**. List of all the input trees taken from primary literature and used in the supertree analysis. A matrix indicating any remaining data non-independence is provided. Click here for file

Additional file 3 **Software and settings for supertree analyses**. Details of software and settings used for different supertree methods. References provided. Click here for file

Additional file 4 **V and V+ scores for extended majority rule Hymenoptera supertrees**. All relationships recovered by each supertree analysis provided with V and V+ score, number of supporting and conflicting input trees. Click here for file

Additional file 5 **Overall V scores for all Hymenoptera supertrees**. For each supertree constructed as part of this study an overall V (and V+) score is provided, along with an indication of how complete the supertree is and how resolved the supertree is. Where certain taxa are omitted, these are indicated. Click here for file

Additional file 6 **Other shifts detected in compartmentalised MRC tree**. A list of the shifts detected in the compartmentalised MRC supertree not detected in the all-inclusive MRC supertree is provided. Click here for file

Additional file 7 **MRC supertree of hymenopteran families from a compartmentalised analysis in which Apocrita and Aculeata are constrained as monophyletic**. Further description N/A. Click here for file

Additional file 8 **Standard MRP supertree of hymenopteran families from a compartmentalised analysis in which Apocrita and Aculeata are constrained as monophyletic**. Further description N/A. Click here for file

Additional file 9 **Sister group species richness comparisons**. All sister group species richness comparisons as carried out using the method of Davies et al. (2004). Explanation of analysis provided. Click here for file
