# Supplementary material for: The origins of species richness in the Hymenoptera: insights from a family-level supertree
Source: BMC Evol Biol. 2010 Apr 27;10:109. doi: 10.1186/1471-2148-10-109 (PMC2873417; doi:10.1186/1471-2148-10-109)
Supplement: Additional file 1 — List of valid hymenopteran families. A list of all families recognised as valid for supertree analysis, information on whether they could be included in the analysis or not, and synonyms also provided. [file 1471-2148-10-109-S1.PDF]

ADDITIONAL FILE 1: LIST OF VALID HYMENOPTERAN FAMILIES

Valid Hymenoptera Families<br>Gordh & Headrick (2000)<br>Ross & Jarzembowski (1993)<br>EDNA Fossil Database	Recognised Synonyms	Included in supertree analysis?	Extant family taxonomy<br>In agreement unless stated<br>Gauld & Bolton 1988	Goulet & Huber 1993
Adrenidae	Andrenidae		subfamily of Apidae	
Agaonidae	Agaontidae			
Agriotypidae			?	subfamily of Ichneumonoidea
Alloxystidae			subfamily of Charipidae	subfamily of Charipidae
Ampulicidae			?	
Anarchitidae			subfamily of Figitidae	subfamily of Figitidae
Anaxyelidae	Syntexidae			
Andreneliidae*				
Anomopterellidae*				
Anthophoridae			subfamily of Apidae	
Aphelinidae				
Aphidiidae			subfamily of Braconidae	subfamily of Braconidae
Apidae	Bombidae			
Apozygidae			subfamily of Ichneumonoidea	subfamily of Braconidae
Archaeocynipidae*				
Archimymenidae			?	?
Argidae				
Aulacidae				
Austroniidae				
Austroserphidae			subfamily of Proctotrupidae	subfamily of Proctotrupidae
Baissidae			?	?
Baissodidae*				
Bethylidae				
Bethylonymidae*				
Blasticotomidae				
Braconidae	Branconidae			
Bradynobaenidae				
Cephidae				
Ceraphronidae				
Chalcididae	Chalcidae			
Charipidae				
Chrysididae				
Cimbicidae				
Colletidae			subfamily of Apidae	
Cretevaniidae*				
Ctenoplectidae			?	
Cynipidae				
Daohugoidae*				
Diapriidae				
Diprionidae				
Dryinidae				
Elasmidae				
Electrotomidae*				
Embolemidae				
Encyrtidae				
Eoichneumonidae*				
Ephialtitidae*	Karataidae			
Eucharitidae				

Eucoilidae			
Eulophidae	Aphelidae		
Eumenidae			subfamily of Vespidae
Eupelmidae			
Eurytomidae			
Evaniidae			
Falsiformicidae*			
Fideliidae		?	
Figitidae			
Formicidae	Dolichoderidae, Palaeosminthuridae		
Gasteruptiidae			
Halictidae		subfamily of Apidae	
Heloridae			
Heterogynidae	Heterogynaidae	?	
Ibaliidae			
Ichneumonidae	Myersiidae		
Jurapriidae*			
Karatavitidae*			
Khutelchalcididae*			
Konowiellidae		?	?
Kotujellidae		?	?
Leucospidae			
Limnetidae*			
Liopteridae			
Loboscelidiidae		subfamily of Chrysididae	subfamily of Chrysididae
Maimetshidae*			
Masaridae			subfamily of Vespidae
Megachilidae		subfamily of Apidae	
Megalodontidae			
Megalyridae	Megaliridae		
Megaspilidae			
Melittidae		subfamily of Apidae	
Mesoserphidae		?	?
Monomachidae			
Mutillidae			
Mymaridae			
Mymarommatidae			
Ormyridae			
Orussidae	Oryssidae		
Oxaeidae		subfamily of Apidae	
Palaeocynipidae*			
Paleomelittidae*			
Pamphiliidae			
Pararhophitidae		?	
Paroryssidae*	Parorysidae		
Paxylommatidae	Hybrizontidae	subfamily of Ichneumonoidea	subfamily of Ichneumonoidea
Pelecinidae	Pelecinopteridae		
Peradeniidae			
Pergidae			
Perilampidae			
Platygasteridae	Platygastridae		
Plumariidae			
Pompillidae	Pompilidae, Psammocharidae		

Praeaulacidae*		Yes - but only included in strict concensus tree		
Praeichneumonidae*		Yes - in extended majority rule tree		
Praesiricidae*		Yes - but only included in strict concensus tree		
Proctotrupidae	Serphitidae	Yes - in extended majority rule tree		
Pteromalidae	Cleonymidae	Yes - in extended majority rule tree		
Pterygophoridae		Yes - in extended majority rule tree	?	?
Rhopalosomatidae		Yes - in extended majority rule tree		
Roproniidae		Yes - in extended majority rule tree		
Rotoitidae		No - absent from input trees		
Sapygidae	Cossilidae, Fedtschenkidae	Yes - in extended majority rule tree		
Scelionidae		Yes - in extended majority rule tree		
Scenopinidae	Omphralidae	No - absent from input trees	?	?
Sclerogibbidae		Yes - in extended majority rule tree		
Scolebythidae		Yes - in extended majority rule tree		
Scoliidae		Yes - in extended majority rule tree		
Sepulcidae*		Yes - in extended majority rule tree		
Serphitidae*		Yes - but only included in strict concensus tree		
Sierolomorphidae		Yes - in extended majority rule tree		
Signiphoridae		Yes - in extended majority rule tree		
Siricidae	Sinosiricidae	Yes - in extended majority rule tree		
Sphecidae	Angarosphecidae, Pemphredonidae, Trypoxylidae, Larridae, Crabronidae, Philanthidae	Yes - in extended majority rule tree		
Sphecomyrmidae*		No - absent from input trees		
Stenotritidae		Yes - in extended majority rule tree	?	
Stephanidae		Yes - in extended majority rule tree		
Stigmaphronidae*		Yes - in extended majority rule tree		
Stolamissidae*		Yes - in extended majority rule tree		
Tanaostigmatidae		Yes - in extended majority rule tree		
Tenthredinidae		Yes - in extended majority rule tree		
Tetracampidae		Yes - in extended majority rule tree		
Thynnidae		No - absent from input trees	?	subfamily of Tiphiidae
Tiphiidae		Yes - in extended majority rule tree		
Torymidae	Callimomidae	Yes - in extended majority rule tree		
Trichogrammatidae		Yes - in extended majority rule tree		
Trigonalidae	Ichneumonomimidae	Yes - in extended majority rule tree		
Trupochalcididae		No - absent from input trees	?	?
Vanhorniidae		Yes - in extended majority rule tree		
Vespidae		Yes - in extended majority rule tree		
Xiphydriidae		Yes - in extended majority rule tree		
Xyelidae		Yes - in extended majority rule tree		
Xyelotomidae*		Yes - in extended majority rule tree		
Xyelydidae*	Xyelididae	Yes - but only included in strict concensus tree		

Yes - in extended majority rule tree
Yes - but only included in strict concensus tree
No - absent from input trees
