# Supplementary material for: The origins of species richness in the Hymenoptera: insights from a family-level supertree
Source: BMC Evol Biol. 2010 Apr 27;10:109. doi: 10.1186/1471-2148-10-109 (PMC2873417; doi:10.1186/1471-2148-10-109)
Supplement: Additional file 2 — Input trees and data non-independence. List of all the input trees taken from primary literature and used in the supertree analysis. A matrix indicating any remaining data non-independence is provided. [file 1471-2148-10-109-S2.PDF]

ADDITIONAL FILE 2: INPUT TREES AND DATA NON-INDEPENDENCE

Input Trees

Author	Year	Tree
Alexander	1992	Fig10
Alexander & Michener	1995	Fig5
Alexander & Michener	1995	Fig6
Alexander & Michener	1995	Fig10
Alexander & Michener	1995	Fig12
Alexander & Michener	1995	Fig13
Alexander & Michener	1995	Fig14
Alexander & Michener	1995	Fig15
Brothers	1999	Fig6
Campbell et al	2000	Fig1
Carpenter & Wheeler	1999	Fig2
Carpenter & Wheeler	1999	Fig3
Castro & Dowton	2006	Fig2
Danforth et al	2006	Fig3
Danforth et al	2006	Fig4
Danforth et al	2006	Fig5
Dowton & Austin	1997	Fig1
Dowton & Austin	1997	Fig3
Dowton & Austin	2001	Fig2
Dowton & Austin	2001	Fig3
Dowton & Austin	2001	Fig4
Dowton & Austin	2001	Fig5
Dowton & Austin	2001	Fig6
Dowton & Austin	2001	Fig7
Dowton & Austin	2001	Fig8
Dowton & Austin	2001	Fig9
Dowton & Austin	2001	Fig10
Dowton & Austin	2001	Fig11
Dowton & Austin	2001	Fig12
Dowton & Austin	2001	Fig13
Dowton et al	1997	Fig1
Engel	2001	Fig122x123
Konigsmann	1977x1978ax1978b	Fig6xText(Symphyta)xFig7axText(Parasitica)xFig13xText(Aculeata)
Konigsmann	1977x1978ax1978b	Fig6xText(Symphyta)xFig7bxText(Parasitica)xFig13xText(Aculeata)
Konigsmann	1977x1978ax1978b	Fig6xText(Symphyta)xFig7cxText(Parasitica)xFig13xText(Aculeata)
Krogmann & Vilhelmsen	2006	Fig21
Krogmann & Vilhelmsen	2006	Fig22
Krogmann & Vilhelmsen	2006	Fig23
Liu et al	2007	Fig21/Fig25
Melo	1999	Fig3
Melo	1999	Fig4a
Quicke et al	1999	Fig13
Ronquist et al	1999	Fig2x6x12
Ronquist et al	1999	Fig2x6x13
Ronquist et al	1999	Fig3x6x12
Ronquist et al	1999	Fig3x6x13
Schulmeister	2003a	Fig7
Schulmeister	2003a	Fig7 (Apocrita)
Schulmeister	2003b	Fig1
Schulmeister	2003b	Fig2
Schulmeister et al	2002	Fig2a
Schulmeister et al	2002	Fig2b
Schulmeister et al	2002	Fig2c
Schulmeister et al	2002	Fig2d
Schulmeister et al	2002	Fig3 1:1:1
Schulmeister et al	2002	Fig3 2:2:1
Schulmeister et al	2002	Fig3 4:4:1
Schulmeister et al	2002	Fig3 2:1:1
Schulmeister et al	2002	Fig3 4:2:1
Schulmeister et al	2002	Fig3 8:4:1
Schulmeister et al	2002	Fig3 8:2:1
Schulmeister et al	2002	Fig3 16:4:1
Schulmeister et al	2002	Fig3 4:1:1(left)
Schulmeister et al	2002	Fig3 4:1:1(right)
Schulmeister et al	2002	Fig5 1:1:1
Schulmeister et al	2002	Fig5 2:2:1
Schulmeister et al	2002	Fig5 4:4:1
Schulmeister et al	2002	Fig5 2:1:1
Schulmeister et al	2002	Fig5 4:2:1
Schulmeister et al	2002	Fig5 8:4:1
Schulmeister et al	2002	Fig5 4:1:1
Schulmeister et al	2002	Fig5 8:2:1
Schulmeister et al	2002	Fig5 16:4:1
Serrao	2005	Fig15
Wheeler et al	2001	Fig12a
Wheeler et al	2001	Fig14
Whiting	2002	Fig4

Input Tree References for Final Data Set

Alexander, B. 1992. An exploratory analysis of cladistic relationships within the superfamily Apoide with special reference to sphecid wasps (Hymenoptera). *Journal of Hymenopteran Research* **1**: 25-61.

Alexander, B. & Michener, C. D. 1995. Phylogenetic studies of the families of short-tongued bees (Hymenoptera: Apoidea). *The University of Kansas Science Bulletin* **55**: 377-424.

Brothers, D. J. 1999. Phylogeny and evolution of wasps, ants and bees (Hymenoptera, Chrysidoidea, Vespoidea and Apoidea). *Zoologica Scripta* **28**: 233-249.

Campbell, B., Heraty, J., Rasplus, J.-Y., Chan, K., Steffen-Campbell, J. & Babcock, C. (2000) Molecular systematics of the Chalcidoidea using 28S-D2 rDNA. In: *Hymenoptera: Evolution, Biodiversity and Biological Control*, (Austin, A. D. & Dowton, M., eds.). pp. 59-71. CSIRO Publishing, Collingwood, Australia.

Carpenter, J. M. & Wheeler, W. C. 1999. Towards simultaneous analysis of morphological and molecular data in Hymenoptera. *Zoologica Scripta* **28**: 251-260.

Castro, L. R. & Dowton, M. 2006. Molecular analyses of the Apocrita (Insecta: Hymenoptera) suggest that the Chaclidoidea are sister to the diaprioid complex. *Invertebrate Systematics* **20**: 603-614.

Danforth, B. N., Fang, J. & Sipes, S. 2006. Analysis of family-level relationships in bees (Hymenoptera: Apiformes). *Molecular Phylogenetics and Evolution* **39**: 358-372.

Dowton, M. & Austin, A. D. 1997. Evidence for AT-transversion bias in wasp (Hymenoptera: Symphyta) mitochondrial genes and its implications for the origin of parasitism. *Journal of Molecular Evolution* **44**: 398-405.

Dowton, M. & Austin, A. D. 2001. Simultaneous analysis of 16S, 28S, COI and morphology in the Hymenoptera: Apocrita - evolutionary transitions among parasitic wasps. *Biological Journal of the Linnean Society* **74**: 87-111.

Dowton, M., Austin, A. D., Dillon, N. & Bartowsky, E. 1997. Molecular phylogeny of the apocritan wasps: the Proctotrupomorpha and Evaniomorpha. *Systematic Entomology* **22**: 245-255.

Engel, M. S. 2001. A monograph of the Baltic amber bees and evolution of the Apoidea (Hymenoptera). *Bulletin of the American Museum of Natural History* **259**: 1-192.

Königsmann, E. 1977. Das phylogenetische System der Hymenoptera Teil 2: Symphyta. *Deutsche Entomologische Zeitschrift Neue Folge* **24**: 1-40.

Königsmann, E. 1978a. Das phylogenetische Suystem der Hymenoptera Teil3: Terebrantes (Unterordnung Apocrita). *Deutsche Entomologische Zeitschrift Neue Folge* **25**: 1-55.

Königsmann, E. 1978b. Das phylogenetische System der Hymenoptera Teil 4: Aculeata (Unterordnung Apocrita). *Deutsche Entomologische Zeitschrift Neue Folge* **25**: 365-435.

Krogmann, L. & Vilhelmsen, L. 2006. Phylogenetic implications of the mesosomal skeleton in Chalcidoidea (Hymenoptera, Apocrita) - tree searches in a jungle of homoplasy. *Invertebrate Systematics* **20**: 615-674.

Liu, Z., Engel, M. S. & Grimaldi, D. A. 2007. Phylogeny and geological history of the cynipoid wasps (Hymenoptera, Cynipoidea). *American Museum Novitates* **3583**: 1-48.

Melo, G. A. R. 1999. Phylogenetic relationships and classificationof the major lineages of Apoidea (Hymenoptera) with emphasis on the crabonid wasps. *University of Kansas Natural History Museum Scientific Papers* **14**: 1-55.
Quicke, D. L. J., Basibuyuk, H. H., Fitton, M. G. & Rasnitsyn, A. P. 1999. Morphological, palaeontological and molecular aspects of ichneumonoid phylogeny (Hymenoptera,Insecta). *Zoologica Scripta* **28**: 175-202.
Ronquist, F., Rasnitsyn, A. P., Roy, A., Eriksson, K. & Lindgren, M. 1999. Phylogeny of the Hymenoptera: a cladisitic reanalysis of Rasnitsyn's (1988) data. *Zoologica Scripta* **28**: 13-50.
Schulmeister, S. 2003a. Review of morphological evidence on the phylogeny of basal Hymenoptera (Insecta) with a discussion of the ordering of characters. *Biological Journal of the Linnean Society* **79**: 209-243.
Schulmeister, S. 2003b. Simultaneous analysis of basal Hymenoptera (Insecta): introducing robust-choice sensitivity analysis. *Biological Journal of the Linnean Society* **79**: 245-275.
Schulmeister, S., Wheeler, W. C. & Carpenter, J. M. 2002. Simultaneous analysis of the basal lineages of Hymenoptera (Insecta) using sensitivity analysis. *Cladistics* **18**: 455-484.
Serrão, J. E. 2005. Proventricular structure in solitary bees (Hymenoptera: Apoidea). *Organisms, Diversity and Evolution* **5**: 125-133.
Wheeler, W. C., Whiting, M., Wheeler, Q. D. & Carpenter, J. M. 2001. The phylogeny of the extant hexapod orders. *Cladistics* **17**: 113-169.
Whiting, M. F. 2002. Phylogeny of the holometabolous insect orders: molecular evidence. *Zoologica Scripta* **31**: 3-15.

HYMENOPTERA DATA NON-INDEPENDENCE

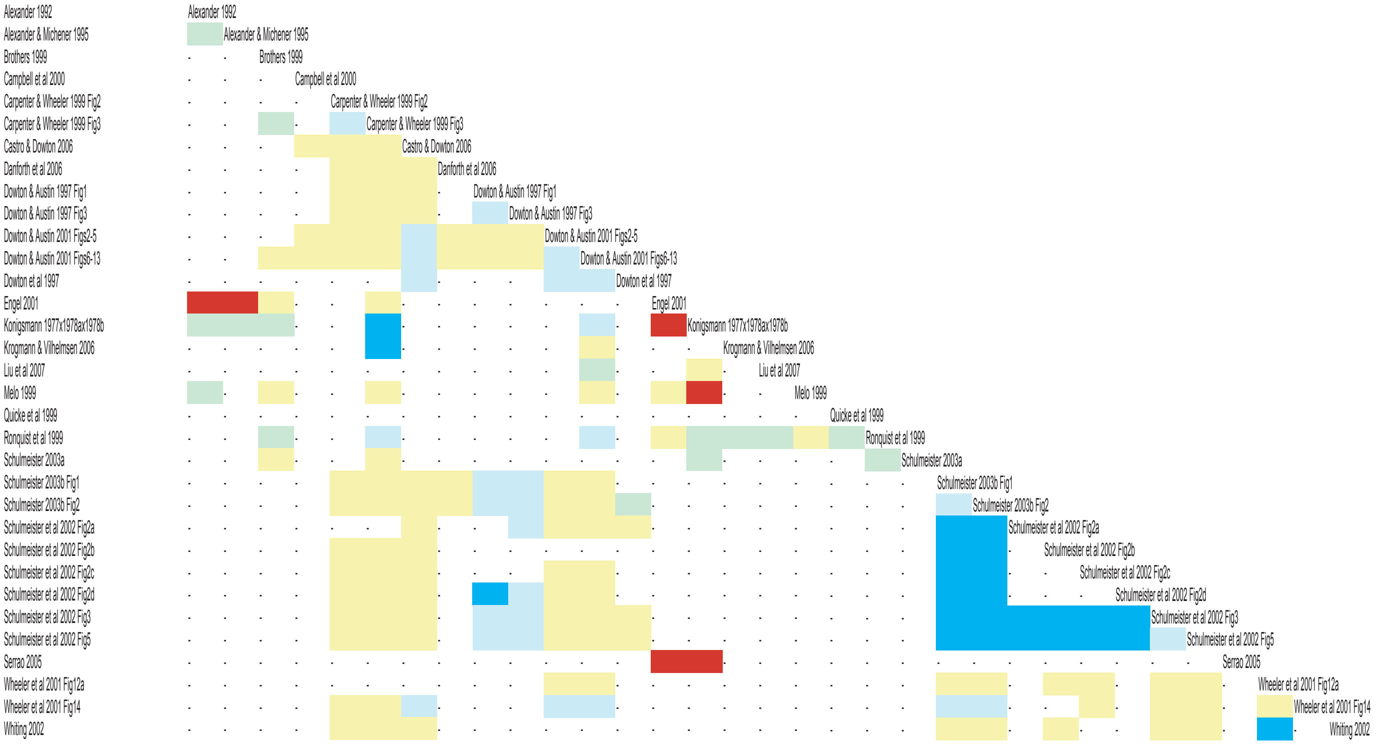

If no taxon and/or data overlap (incudes where nonreplicable studies do not mention characters) = -

<50% taxon and data overlap

>50% taxon overlap but <50% data overlap

>50% taxon overlap and genes but used in combination with different genes and/or morphological data

>50% taxon (order) overlap and gene(s) combination but different gene region (primers used) or species combination

Overlap uncertain and possibly >50%

Takes into account that taxa may have been pruned out of trees and this can reduce data overlap
