# Supplementary material for: The origins of species richness in the Hymenoptera: insights from a family-level supertree
Source: BMC Evol Biol. 2010 Apr 27;10:109. doi: 10.1186/1471-2148-10-109 (PMC2873417; doi:10.1186/1471-2148-10-109)
Supplement: Additional file 3 — Software and settings for supertree analyses. Details of software and settings used for different supertree methods. References provided. [file 1471-2148-10-109-S3.PDF]

ADDITIONAL FILE 3: SOFTWARE AND SETTINGS FOR SUPERTREE ANALYSIS

**Method**	**Software**	**Settings other than defaults**	**Software author (related reference)**
Standard MRP	PAUP* v. 4	Replicates = 100, addition sequence = random	Swofford (2002)
MRC	MIX (PHYLIP v. 3.66)	Replicates = 100, threshold = 2.0 (as for Ross & Rodrigo, 2004)	Felsenstein (1989)
Average Consensus	Clann v. 3.0.2	-	Creevey & McInerney (2005)

Creevey, C. & McInerney, J. O. 2005 Clann: investigating pylogenetic information through supertree analyses. *Bioinformatics* **21**, 390-392.

Felsenstein, J. 1989 PHYLIP -- Phylogeny Inference Package. *Cladistics* **5**, 164-166.

Swofford, D. L. 2002 PAUP*, Phylogenetic Analysis Using Parsimony (*and Other Methods). Sunderland: Sinauer.
