# Supplementary material for: The origins of species richness in the Hymenoptera: insights from a family-level supertree
Source: BMC Evol Biol. 2010 Apr 27;10:109. doi: 10.1186/1471-2148-10-109 (PMC2873417; doi:10.1186/1471-2148-10-109)
Supplement: Additional file 4 — V and V+ scores for extended majority rule Hymenoptera supertrees. All relationships recovered by each supertree analysis provided with V and V+ score, number of supporting and conflicting input trees. [file 1471-2148-10-109-S4.PDF]

ADDITIONAL FILE 4: V AND V+ SCORES FOR EXTENDED MAJORITY RULE HYMENOPTERA SUPERTREES

Superfamilies recovered by at least one analysis as monophyletic are colour-coded, as are relationships within. Green boxes indicate in which supertrees specific clades are recovered. V scores and supporting/conflicting input trees are for MRC trees unless relationship appears only in MRP tree. Because some input trees are upweighted the number of supporting/conflicting input trees exceeds 77 (the final input tree set). Permitting input trees not shown.

Clade	MRC All-inclusive	MRC Compart-mentalised	Standard MRP	V	V+	Supporting Input Trees	Conflicting Input Trees
Hymenoptera	✓	✓	✓	1	1	2376	0
Hymenoptera (excl. Xyelidae)	✓	✓	✓	0.481	0.576	480	168
Hymenoptera (excl. Xyelidae, Tenthredinoidea)	✓	✓	✓	0.111	0.111	640	512
Tenthredinoidea	✓	✓	✓	0.556	0.694	616	176
Tenthredinoidea (excl. Xyelotomidae)	✓		✓	0.374	0.569	544	248
Tenthredinoidea (excl. Blasticotomidae)		✓		0.418	0.604	556	228
Tenthredinoidea (excl. Xyelotomidae, Blasticotomidae)	✓	✓	✓	0.562	0.729	556	156
Tenthredinoidea (excl. Xyelotomidae, Blasticotomidae, Electrotomidae)		✓		0.461	0.667	520	192
Argidae + Electrotomidae + Pergidae + Pterygophoridae	✓		✓	0.871	0.929	520	36
Argidae + Pergidae + Pterygophoridae	✓	✓	✓	0.871	0.929	520	36
Argidae + Pergidae	✓	✓		1	1	484	0
Argidae + Pterygophoridae			✓	0	0.907	36	36
Cimbicidae + Diprionidae + Tenthredinidae	✓	✓	✓	0.051	0.517	248	224
Cimbicidae + Diprionidae	✓			-0.48	0.245	104	296
Cimbicidae + Tenthredinidae		✓	✓	-0.2	0.372	144	216
Hymenoptera (excl. Xyelidae, Tenthredinoidea, Megalodontoidea)	✓	✓	✓	0.028	0.028	592	560
Megalodontoidea				0.303	0.361	516	276
Hymenoptera (excl. Xyelidae, Tenthredinoidea, Megalodontoidea, Cephoidea)	✓	✓	✓	-0.176	-0.176	504	720
Cephoidea	✓	✓	✓	1	1	72	0
Hymenoptera (excl. Xyelidae, Tenthredinoidea, Megalodontoidea, Cephoidea, Xipydriidae)			✓	-0.847	-0.739	88	1064
Hymenoptera (excl. Xyelidae, Tenthredinoidea, Megalodontoidea, Cephoidea, Siricoidea)	✓	✓		-0.5	-0.412	288	864
Siricoidea	✓	✓	✓	-0.27	0.316	184	320
Apocrita + Orussoidea	✓	✓	✓	-0.733	-0.529	144	936
Apocrita (excl. Karatavitidae) + Orussoidea	✓			-0.733	-0.529	144	936
Apocrita		✓	✓	-0.329	-0.292	376	744
Orussoidea	✓	✓	✓	1	1	72	0
Apocrita (excl. Karatavitidae)	✓	✓	✓	-0.329	-0.292	376	744
Apocrita (excl. Karatavitidae, Ephialtitdae)	✓	✓	✓	-0.329	-0.292	376	744
Apocrita (excl. Karatavitidae, Ephialtitdae, Praeichneumonidae, Stolamissidae, Megalyridae, Eurytomidae)		✓		-0.591	-0.487	328	1278

Praeichneumonidae + Stolamissidae + Megalyridae + Eurytomidae	0	1	0	0
Megalyridae + Eurytomidae	0	1	0	0
Apocrita (excl. Karatavitidae, Ephialtitdae, Praeichneumonidae, Stolamissidae, Megalyridae, Eurytomidae, Ceraphronoidea, Evanioidea)	-0.594	-0.565	344	1352
Trigonalidae + Ichneumonoidea + Platygasteridae + Scelionidae + Cynipoidea + Mymarommatoidea + Chalcidoid (excl. Eurytomidae) & Proctotrupoid Families	-0.602	-0.2	256	1032
Ichneumonoidea + Platygasteridae + Scelionidae + Cynipoidea + Mymarommatoidea + Chalcidoid (excl. Eurytomidae) & Proctotrupoid Families	-0.602	0.204	156	627
Platygasteridae + Scelionidae + Cynipoidea + Mymarommatoidea + Chalcidoid (excl. Eurytomidae) & Proctotrupoid Families	-0.248	0.589	177	294
Platygasteridae + Scelionidae + Cynipoidea + Pelecinidae + Roproniidae	-0.583	0.402	90	342
Platygasteridae + Scelionidae + Cynipoidea	-0.643	0.276	90	414
Platygasteridae + Scelionidae	1	1	243	0
Apocrita (excl. Karatavitidae, Ephialtitdae, Ceraphronoidea, Evanioidea)	-0.708	-0.672	216	1264
Praeichneumonidae + Ichneumonoidea + Platygasteridae + Scelionidae + Cynipoidea + Mymarommatidae + Chalcidoid & Proctotrupoid Families	-0.788	0.254	60	507
Ichneumonoidea + Platygasteridae + Scelionidae + Cynipoidea + Mymarommatidae + Chalcidoid & Proctotrupoid Families	-0.587	0.292	132	507
Platygasteridae + Scelionidae + Cynipoidea + Mymarommatidae + Chalcidoid & Proctotrupoid Families	-0.064	0.73	153	174
Platygasteridae + Scelionidae + Cynipoidea + Mymarommatidae + Diapriidae + Monomachidae + Chalcidoid Families	-1	0.371	0	405
Roproniidae + Pelecinidae + Heloridae + Proctotrupidae + Vanhorniidae	-0.632	-0.292	63	279
Mymarommatidae + Diapriidae + Monomachidae + Chalcidoid Families	-0.208	0.341	171	261
Mymarommatidae + Chalcidoid Families	1	1	360	0
Mymarommatidae + Chalcidoid Families (excl. Eurytomidae, Leucospidae, Chalcididae)	-0.156	0.35	171	234
Mymarommatidae + Chalcidoid Families (excl. Eurytomidae, Leucospidae, Chalcididae, Eulophidae, Perilampidae, Eucharitidae)	-0.052	0.408	192	213
Eulophidae + Perilampidae + Eucharitidae	0	0.6	72	72
Mymarommatidae + Praeichneumonidae + Stolamissidae + Cynipoid, Chalcidoid, Platygasteridae + Scelionidae & Proctotrupoid Families	-0.064	0.744	153	174
Roproniidae + Pelecinidae + Praeichneumonidae + Stolamissidae + Tanaostigmatidae + Platygasteridae + Scelionidae & Cynipoid Families	-0.5	0.556	90	270
Roproniidae + Pelecinidae	-0.234	-0.208	162	261
Praeichneumonidae + Stolamissidae + Tanaostigmatidae + Platygasteridae + Scelionidae + Cynipoid Families	-0.583	0.438	90	342
Praeichneumonidae + Stolamissidae + Tanaostigmatidae + Platygasteridae + Scelionidae	0.542	0.771	243	72
Praeichneumonidae + Stolamissidae	0	1	0	0
Tanaostigmatidae + Platygasteridae + Scelionidae	1	1	243	0
Scelionidae + Tanaostigmatidae	0	1	0	0
Cynipoidea	0.714	0.874	432	72
Cynipoidea (excl. Liopteridae)	0.667	0.874	360	72
Cynipoidea (excl. Archaeocynipidae)	0.714	0.874	432	72
Figitidae + Cynipidae + Ibaliidae	0.667	0.874	360	72
Cynipidae + Ibaliidae	0.316	0.795	225	117

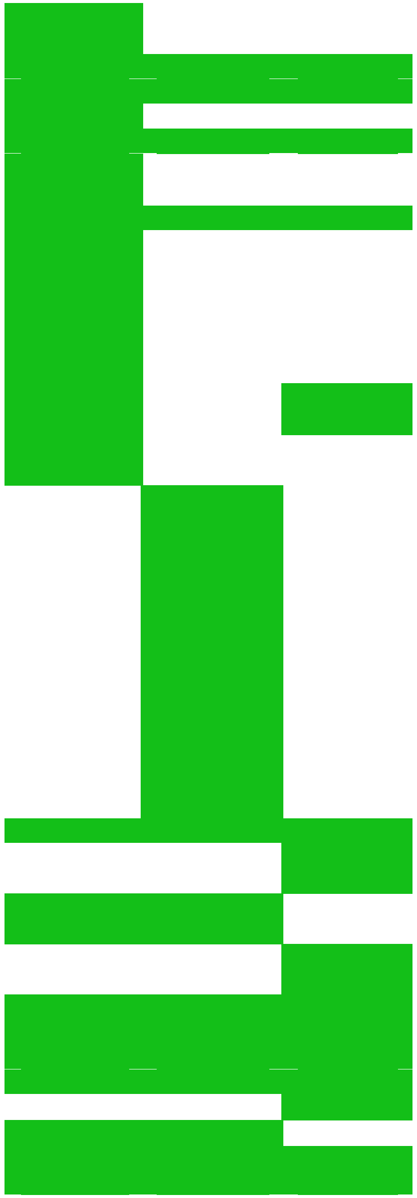

Heloridae + Vanhorniidae + Proctotrupidae + Monomachidae + Diapriidae + Mymarommatidae + Chalcidoid Families (excl. Tanaostigmatidae)	-0.667	0.167	72	360
Heloridae + Vanhorniidae + Proctotrupidae	-0.292	-0.292	153	279
Vanhorniidae + Proctotrupidae	0.6	0.667	288	72
Monomachidae + Diapriidae + Mymarommatidae + Chalcidoid Families (excl. Tanaostigmatidae)	-0.322	0.229	171	333
Monomachidae + Diapriidae	0.179	0.319	207	144
Mymarommatidae + Chalcidoid Families (excl. Tanaostigmatidae)	0.667	0.833	360	72
Eucharitidae + Perilampidae + Tetracampidae + Elasmidae + Eulophidae	0	0.6	72	72
Eucharitidae + Perilampidae	1	1	72	0
Tetracampidae + Elasmidae + Eulophidae	1	1	72	0
Elasmidae + Eulophidae	1	1	72	0
Mymarommatidae + Chalcidoid Families (excl. Tanaostigmatidae, Eucharitidae, Perilampidae, Tetracampidae, Elasmidae, Eulophidae)	0.098	0.558	252	207
Mymarommatidae + Chalcidoid Families (excl. Tanaostigmatidae, Eucharitidae, Perilampidae, Tetracampidae, Elasmidae, Eulophidae, Pteromalidae)	-0.07	0.477	180	207
Eurytomidae + Chalcididae + Leucospidae	0.333	0.926	48	24
Chalcididae + Leucospidae	0.333	0.926	48	24
Mymarommatidae + Chalcidoid Families (excl. Tanaostigmatidae, Eucharitidae, Perilampidae, Tetracampidae, Elasmidae, Eulophidae, Pteromalidae, Eurytomidae, Chalcididae, Leucospidae)	-0.279	0.26	120	213
Heloridae + Proctotrupidae + Vanhorniidae + Monomachidae + Diapriidae + Mymarommatidae + Chalcidoid Families (excl. Eurytomidae)	-0.556	0.222	96	336
Monomachidae + Diapriidae + Mymarommatidae + Chalcidoid Families (excl. Eurytomidae)	-0.226	0.285	195	309
Mymarommatidae + Chalcidoid Families (excl. Eurytomidae)	0.778	0.889	384	48
Mymarommatidae + Chalcidoid Families (excl. Eurytomidae, Tanaostigmatidae)	0.524	0.744	384	120
Mymarommatidae + Chalcidoid Families (excl. Eurytomidae, Tanaostigmatidae, Leucospidae, Perilampidae, Eucharitidae, Tetracampidae, Elasmidae, Eulophidae, Pteromalidae)	-0.07	0.425	180	207
Leucospidae + Perilampidae + Eucharitidae + Tetracampidae + Elasmidae + Eulophidae + Pteromalidae	0	0.778	72	72
Perilampidae + Eucharitidae + Tetracampidae + Elasmidae + Eulophidae + Pteromalidae	0	0.75	72	72
Tetracampidae + Elasmidae + Eulophidae + Pteromalidae	1	1	72	0
Elasmidae + Eulophidae + Pteromalidae	1	1	72	0
Eulophidae + Pteromalidae	0	1	0	0
Eupelmidae + Encyrtidae	0.846	0.9	216	18
Mymarommatidae + Aphelinidae + Trichogrammatidae + Signiphoridae + Mymaridae + Agaonidae + Ormyridae + Eupelmidae + Encyrtidae	-0.28	0.26	120	213
Mymarommatidae + Aphelinidae + Trichogrammatidae + Signiphoridae + Mymaridae + Agaonidae + Ormyridae + Torymidae	-0.423	0.177	237	243
Mymarommatidae + Aphelinidae + Trichogrammatidae + Signiphoridae + Mymaridae + Agaonidae + Eupelmidae + Encyrtidae	-0.423	0.177	96	237
Mymarommatidae + Aphelinidae + Trichogrammatidae + Signiphoridae + Mymaridae + Agaonidae + Ormyridae	-0.667	0.583	24	120
Mymarommatidae + Aphelinidae + Trichogrammatidae + Signiphoridae + Mymaridae + Agaonidae	-0.6	0.667	24	96
Mymarommatidae + Aphelinidae + Trichogrammatidae + Signiphoridae + Mymaridae	0	0.75	72	72
Mymarommatidae + Aphelinidae + Trichogrammatidae + Mymaridae	-1	0.5	0	144
Mymarommatidae + Aphelinidae + Trichogrammatidae + Signiphoridae	-0.333	0.333	48	96
Mymarommatidae + Aphelinidae + Trichogrammatidae	0.333	0.833	48	24
Aphelinidae + Trichogrammatidae	1	1	48	0

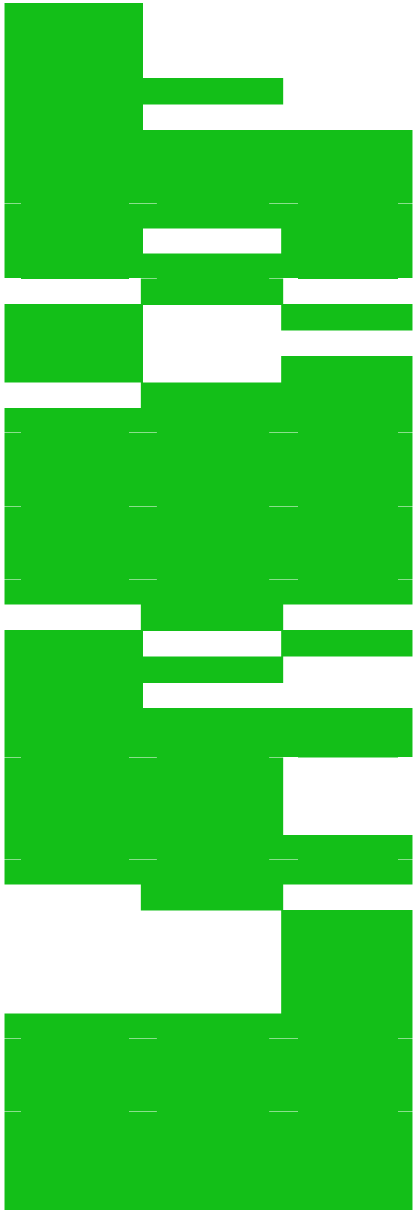

Ichneumonoidea + Ceraphronoidea + Evanioidea + Stephanidae + Trigonalidae + Megalyridae + Aculeata	-0.6	-0.495	296	1184
Ichneumonoidea	0.571	0.75	396	108
Ceraphronoidea + Evanioidea + Stephanidae + Trigonalidae + Megalyridae + Aculeata	-0.669	-0.554	233	1175
Ceraphronoidea + Evanioidea	0.298	0.515	549	297
Ceraphronoidea	1	1	432	0
Megaspilidae + Ceraphronidae	1	1	432	0
Evanioidea	0.418	0.632	549	225
Evanioidea (excl. Gasteruptiidae)	1	1	72	0
Evaniidae + Cretevaniidae	1	1	72	0
Gasteruptiidae + Aulacidae	0	1	0	0
Stephanidae + Trigonalidae + Megalyridae + Aculeata	-0.642	-0.529	252	1156
Stephanidae + Trigonalidae + Megalyridae	-0.347	-0.031	360	742
Trigonalidae + Megalyridae	-0.31	0.311	261	496
Stephanidae + Aculeata	-0.594	-0.565		
Aculeata	0.038	0.342	486	450
Chrysidoidea	0	0.333	216	216
Chrysidoidea (excl. Plumariidae)	0	0.333	216	216
Chrysidoidea (excl. Plumariidae, Scolebythidae)	-0.091	0.333	180	216
Bethylidae + Chrysididae	0.111	0.556	180	144
Dryinidae + Embolemidae + Sclerogibbidae	0	0	108	108
Dryinidae + Embolemidae	0.667	0.667	180	36
Apoidea + Vespoid Families	-0.161	0.188	423	585
Apoidea + Vespoid Families excl. Sierolomorphidae	-0.589	-0.113	207	801
Vespoidea	-0.628	0.125	144	630
Vespoidea excl. Sierolomorphidae	-0.795	0.125	72	630
Vespoidea excl. Sierolomorphidae, Pompillidae, Rhopalosomatidae	-0.795	0.125	72	630
Pompillidae + Rhopalosomatidae	0.25	0.5	180	108
Tiphiidae + Mutillidae + Sapygidae	0	0.6	144	144
Mutillidae + Sapygidae	0.333	0.714	144	72
Bradynobaenidae + Vespidae + Scoliidae + Falsiformicidae + Formicidae	-0.527	0.475	117	378
Vespidae + Scoliidae + Falsiformicidae + Formicidae	-0.527	0.475	117	378
Vespidae + Scoliidae	0	0.789	144	144
Falsiformicidae + Formicidae	0	0.889	36	36
Vespoidea excl. Sierolomorphidae, Tiphiidae, Mutillidae, Sapygidae	-0.633	0.275	117	522
Vespoidea excl. Pompillidae, Rhopalosomatidae	-0.692	0.175	108	594
Sierolomorphidae + Formicidae + Falsiformicidae	-0.5	0.667	36	108
Bradynobaenidae + Vespidae + Scoliidae + Tiphiidae + Mutillidae + Sapygidae	-0.619	0.363	108	459
Bradynobaenidae + Vespidae + Scoliidae	-1	0.684	0	216
Apoidea	0.667	0.818	360	72
Anthophila + Sphecidae + Heterogynaeidae	0.467	0.692	396	144
Anthophila + Sphecidae	0.467	0.692	396	144
Anthophila	1	1	144	0
Anthophila (excl. Colletidae, Stenotritidae)	0.125	0.781	162	126
Colletidae + Stenotritidae	0.5	0.833	108	36
Anthophila (excl. Colletidae, Stenotritidae, Halictidae)	0.167	0.792	168	120
Anthophila (excl. Colletidae, Stenotritidae, Halictidae, Oxaeidae, Adrenidae)	0.556	0.917	168	48

Oxaeidae + Adrenidae	0.5	0.917	54	18
Melittidae + Megachilidae + Apidae + Anthophoridae	0.556	0.917	168	48
Megachilidae + Apidae + Anthophoridae	1	1	216	0
Apidae + Anthophoridae	0	1	0	0
Anthophoridae + Megachilidae	1	1	72	0
