# Supplementary material for: The origins of species richness in the Hymenoptera: insights from a family-level supertree
Source: BMC Evol Biol. 2010 Apr 27;10:109. doi: 10.1186/1471-2148-10-109 (PMC2873417; doi:10.1186/1471-2148-10-109)
Supplement: Additional file 5 — Overall V scores for all Hymenoptera supertrees. For each supertree constructed as part of this study an overall V (and V+) score is provided, along with an indication of how complete the supertree is and how resolved the supertree is. Where certain taxa are omitted, these are indicated. [file 1471-2148-10-109-S5.PDF]

ADDITIONAL FILE 5: OVERALL V SCORES FOR HYMENOPTERA SUPERTREES

Method	All-in (A)/ Compart-mentalised (C)	Strict (S)/ Majority Rule (MR)/ Extended Majority Rule (XMR)	No. Taxa	No. of nodes (= degree of resolution)	V	V+
Standard MRP	C	S	102	46	0.286	0.532
Standard MRP	C	MR	102	88	0.162	0.505
Standard MRP	C	XMR	96	95	0.129	0.502
MRC	C	S	113	49	0.218	0.502
MRC	C	S	102 (as for MRP)	49	0.219	0.504
MRC	C	MR	113	104	0.114	0.536
MRC	C	MR	102 (as for MRP)	94	0.143	0.517
MRC	C	XMR	100	99	0.148	0.522
MRC	C	XMR	96 (as for MRP)	95	0.158	0.516
MRC	A	S	113	78	0.192	0.583
MRC	A	S	102 (as for MRP)	77	0.194	0.577
MRC	A	MR	113	109	0.167	0.553
MRC	A	MR	102 (as for MRP)	99	0.184	0.537
MRC	A	XMR	100	99	0.169	0.530
MRC	A	XMR	96 (as for MRP)	95	0.163	0.514
Average Consensus	C	-	100	99	-0.326	0.098
Average Consensus	A	-	100	99	-0.458	0.099

For MRC extended majority rule trees the 13 taxa removed are:

Austroniidae, Bethylonymidae, Eoichneumonidae, Eumenidae, Jurapriidae, Loboscelidiidae, Maimetshidae, Masaridae, Mesoserphidae, Praeaulacidae, Praesiricidae, Serphitidae, Xyelydidae.

For strict and majority rule analyses the 11 taxa removed MRP analysis are:

Austroniidae, Elasmidae, Eoichneumonidae, Jurapriidae, Maimetshidae, Mesoserphidae, Praeaulacidae, Serphitidae, Stolamissidae, Tetracampidae, Tanaostigmatidae.

Then for extended majority rule MRP trees the other taxa removed are:

Bethylonymidae, Eumenidae, Loboscelidiidae, Masaridae, Praesiricidae, Xyelydidae.
