# Supplementary material for: The origins of species richness in the Hymenoptera: insights from a family-level supertree
Source: BMC Evol Biol. 2010 Apr 27;10:109. doi: 10.1186/1471-2148-10-109 (PMC2873417; doi:10.1186/1471-2148-10-109)
Supplement: Additional file 6 — Other shifts detected in compartmentalised MRC tree. A list of the shifts detected in the compartmentalised MRC supertree not detected in the all-inclusive MRC supertree is provided. [file 1471-2148-10-109-S6.PDF]

ADDITIONAL FILE 6: OTHER SHIFTS DETECTED IN COMPARTMENTALISED MRC TREE

Two-way shift detected in the compartmentalised MRC supertree but one-way in all-inclusive tree:

Hymenoptera (excl. Xyelidae) (+ve), and Xyelidae (–ve)

Shift detected in compartmentalised MRC supertree alone (i.e. where phylogeny conflicts with all-inclusive supertree)

Tenthredinidae (+ve)

Ichneumonoidea + Cynipoidea + Platygasteridae + Scelionidae + Proctotrupoidea + Chalcidoidea (+ve)

Chalcididae + Eupelmidae + Encyrtidae + Torymidae + Ormyridae + Agaonidae + Mymaridae + Signiphoridae + Mymarommatidae + Pteromalidae + Eulophidae + Elasmidae + Tetracampidae + Perilampidae + Eucharitidae + Leucospidae (+ve)

Diapiidae (+ve)
