# Supplementary material for: The origins of species richness in the Hymenoptera: insights from a family-level supertree
Source: BMC Evol Biol. 2010 Apr 27;10:109. doi: 10.1186/1471-2148-10-109 (PMC2873417; doi:10.1186/1471-2148-10-109)

ADDITIONAL FILE 7: MRC SUPERTREE OF HYMENOPTERAN FAMILIES FROM A COMPARTMENTALISED ANALYSIS IN WHICH APOCRITA AND ACULEATA ARE CONSTRAINED AS MONOPHYLETIC

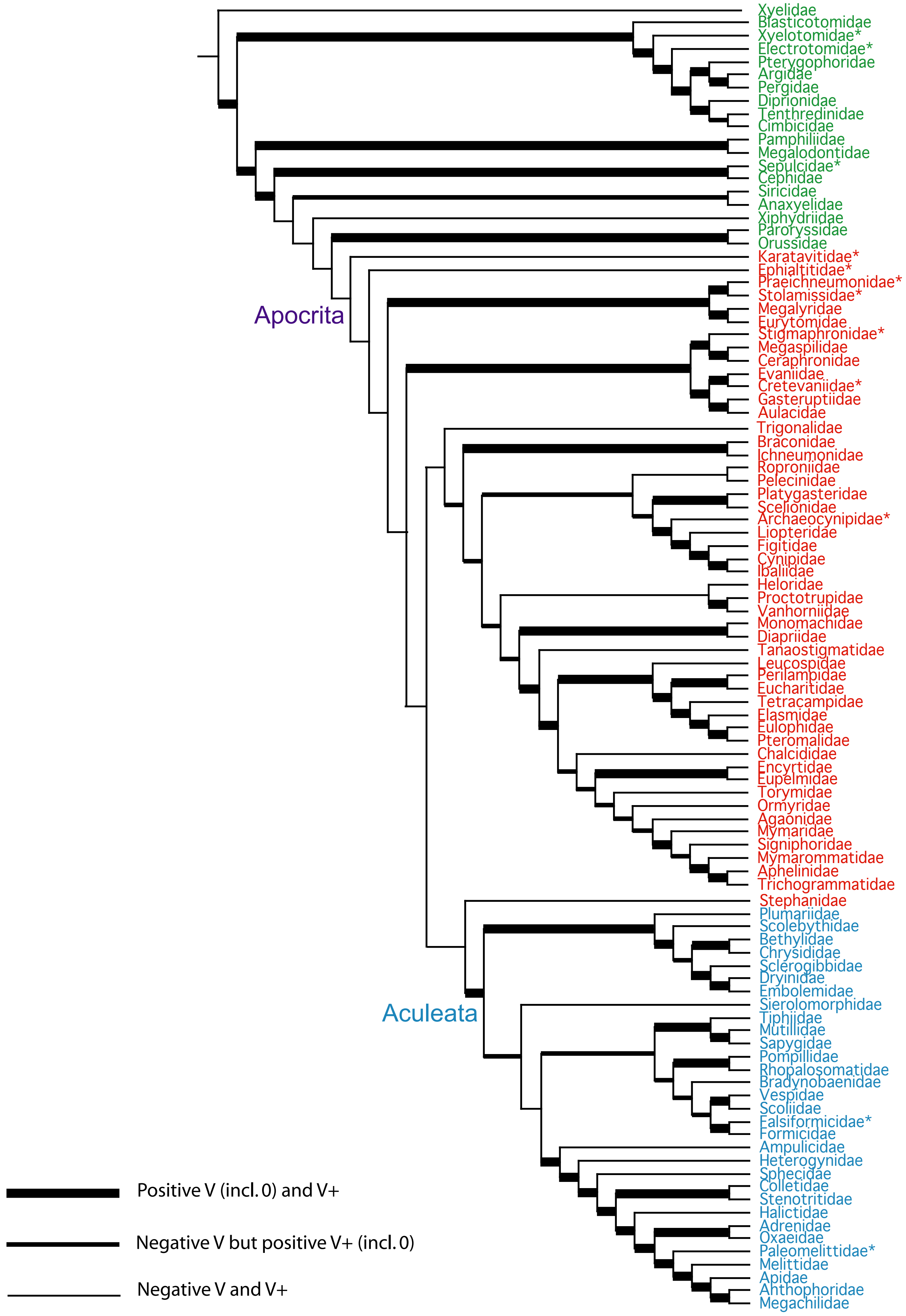

Supplement: Additional file 7 — MRC supertree of hymenopteran families from a compartmentalised analysis in which Apocrita and Aculeata are constrained as monophyletic. Further description N/A. [file 1471-2148-10-109-S7.PDF]
