# Supplementary material for: The origins of species richness in the Hymenoptera: insights from a family-level supertree
Source: BMC Evol Biol. 2010 Apr 27;10:109. doi: 10.1186/1471-2148-10-109 (PMC2873417; doi:10.1186/1471-2148-10-109)
Supplement: Additional file 9 — Sister group species richness comparisons. All sister group species richness comparisons as carried out using the method of Davies et al. (2004). Explanation of analysis provided. [file 1471-2148-10-109-S9.PDF]

ADDITIONAL FILE 9: SISTER GROUP SPECIES RICHNES COMPARISONS

Potential significant shifts are highlighted in green. Pairs of taxa associated with these shifts (labelled A and B) are then subjected to the Davies et al. (2004) trickle-down correction method against their outgroup (C). Where a positive shift is detected this is highlighted in light green, and where a negative shift is detected this is highlighted in pink. The taxon in which a shift has occurred has its species richness corrected before subsequent comparisons at deeper nodes are carried out. In these subsequent comparisons, where a potential shift is detected it is highlighted in green, and is then subjected to the Davies et al. (2004) method itself. There may be cases where comparison with the outgroup (C) does not register a significant shift. In these cases no correction is made.

In the tables below, taxon names between the order and family-level are used to group multiple taxa together as follows:

Higher Taxon Name	Included Families
Anthophila	Stenotritidae, Colletidae, Halictidae, Oxaeidae, Adrenidae, Anthophoridae, Apidae, Megachilidae, Melittidae
Spheciformes	Sphecidae, Heterogynidae, Ampulicidae
Apoidea	Anthophila + Spheciformes
Vespoidea	Sierolomorphidae, Pompilidae, Rhopalosomatidae, Tiphiidae, Mutilidae, Sapygidae Bradynobaenidae, Vespidae, Scoliiidae, Formicidae
Chrysidoidea	Plumariidae, Scolebythidae, Bethylidae, Chrysididae, Sclerogibbidae, Embolemidae, Dryinidae
Aculeata	Apoidea + Vespoidea + Chrysidoidea
Evanioidea	Evaniidae, Gasteruptiidae, Aulacidae
Ceraphronoidea	Ceraphronidae, Megaspilidae
Ichneumonoidea	Ichneumonidae, Braconidae
Platygastroidea	Platygasteridae, Scelionidae
Cynipoidea	Figitidae, Liopteridae, Ibaliidae, Cynipidae
Proctotrupoidea	Diapriidae, Heloridae, Monomachidae, Pelecinidae, Proctotrupidae, Roproniidae, Vanhorniidae
Chalcidoidea	Agaonidae, Aphelinidae, Chalcididae, Elasmidae, Encyrtidae, Eucharitidae, Eulophidae, Eupelmidae, Eurytomidae, Leucospidae, Mymaridae, Ormyridae, Perilampidae, Pteromalidae, Signiphoridae, Tanaostigmatidae, Tetracampidae, Torymidae, Trichogrammatidae
Apocrita	Chalcidoidea, Proctotrupoidea, Cynipoidea, Mymarommatidae, Ichneumonoidea, Ceraphronoidea, Evanioidea, Stephanidae, Trigonalidae, Megalyridae, Aculeata
Megalodontoidea	Megalodontidae, Pamphilidae
Siricoidea	Anaxyelidae, Siricidae
Tenthredinoidea	Blasticotomidae, Diprionidae, Cimbicidae, Tenthredinidae, Argidae, Pergidae, Pterygophoridae

MRC All-inclusive

Smaller Taxon	Species	Larger taxon	Species	p (two-tailed)
Apidae	1000	Anthophoridae	4000	0.400080016
Megachilidae	3000	Anthophoridae + Apidae	5000	0.750093762
Melittidae	100	Anthophoridae + Apidae + Megachilidae	8000	0.024694407

Oxaeidae	20	Adrenidae	2000	0.019811788
These two sister comaprisons are one another's outgroups. Since they cannot be corrected via comparison with an uncorrected outgroup they are combined into one larger clade A comparison of the two species poor lineages with the next outgroup (Halictidae) and then the two species rich lineages with this outgroup is conducted..				
Melittidae & Oxaeidae (A)	120	Halictidae (C)	3500	0.066316662
Halictidae (C)	3500	Adrenidae & Anthophoridae + Apidae + Megachilidae (B)	10000	0.51855693
Halictidae	3500	Oxaeidae + Adrenidae + Anthophoridae + Apidae + Megachilidae + Melittidae	10120	0.513987811
Stenotritidae	30	Colletidae	2000	0.029571217
Stenotritidae (A)	30	Halictidae + Oxaeidae + Adrenidae + Anthophoridae + Apidae + Megachilidae + Melittidae (C)	13620	0.004395926
Colletidae (B)	2000	Halictidae + Oxaeidae + Adrenidae + Anthophoridae + Apidae + Megachilidae + Melittidae (C)	13620	0.256098342
Stenotritidae + Colletidae	4000	Halictidae + Oxaeidae + Adrenidae + Anthophoridae + Apidae + Megachilidae + Melittidae (C)	13620	0.454055281
Sphecidae	6020	Anthophila	17620	0.509327806
Heterogynidae	5	Anthophila + Sphecidae	23640	0.00042294
Heterogynidae (A)	5	Ampulicidae (C)	167	0.058479532
Ampulicidae (C)	167	Anthophila + Sphecidae (B)	23640	0.014030076
Anthophila + Sphecidae + Heterogynidae	10	Ampulicidae	167	0.113636364
Scoliidae	300	Vespidae	800	0.545950864
Scoliidae + Vespidae	4300	Formicidae	8800	0.656538667
Bradynobaenidae	155	Scoliidae + Vespidae + Formicidae	9900	0.030833499
To be compared to Tiphiidae + Sapygidae + Mutillidae, but this clade needs correcting first				
Sapygidae	80	Mutillidae	5000	0.031502264
Sapygidae (A)	80	Tiphiidae (C)	1500	0.101329956
Tiphiidae (C)	1500	Mutillidae (B)	5000	0.461609478
Tiphiidae	1500	Sapygidae + Mutillidae	5080	0.455996352
Bradynobaenidae (A)	155	Sapygidae + Mutillidae + Tiphiidae (C)	6580	0.046035046
Sapygidae + Mutillidae + Tiphiidae (C)	6580	Scoliidae + Vespidae + Formicidae (B)	9900	0.798592148
Sapygidae + Mutillidae + Tiphiidae	6580	Bradynobaenidae + Scoliidae + Vespidae + Formicidae	23000	0.44491024
Rhopalosomatidae	34	Pompillidae	4200	0.016064257
Rhopalosomatidae (A)	34	Bradynobaenidae + Scoliidae + Vespidae + Formicidae + Sapygidae + Mutillidae + Tiphiidae	29580	0.002296289
Pompillidae (B)	4200	Bradynobaenidae + Scoliidae + Vespidae + Formicidae + Sapygidae + Mutillidae + Tiphiidae	29580	0.248675212
Rhopalosomatidae+ Pompillidae	8400	Bradynobaenidae + Scoliidae + Vespidae + Formicidae + Sapygidae + Mutillidae + Tiphiidae	29580	0.44234972
Sierolomorphidae	10	Bradynobaenidae + Scoliidae + Vespidae + Formicidae + Sapygidae + Mutillidae + Tiphiidae + Rhopalosomatidae + Pompillidae	37980	0.000526468
Sierolomorphidae (A)	10	Apoidea (C)	177	0.107526882
Apoidea (C)	177	Bradynobaenidae + Scoliidae + Vespidae + Formicidae + Sapygidae + Mutillidae + Tiphiidae + Rhopalosomatidae + Pompillidae	37980	0.009277702
Vespoidea	20	Apoidea	177	0.204081633

Embolemidae	10	Dryinidae	1100	0.018034265
Embolemidae (A)	10	Sclerogibbidae (C)	10	1.052631579
Sclerogibbidae (C)	10	Dryinidae (B)	1100	0.018034265
Sclerogibbidae	10	Dryinidae + Embolemidae	20	0.689655172
Bethylidae	2200	Chrysididae	3000	0.846316599
Dryinidae + Embolemidae + Sclerogibbidae	30	Bethylidae + Chrysididae	5200	0.011474469
Scolebythidae (C)	3	Dryinidae + Embolemidae + Sclerogibbidae (A)	30	0.1875
Scolebythidae (C)	3	Bethylidae + Chrysididae (B)	5200	0.001153403
Scolebythidae	3	Dryinidae + Embolemidae + Sclerogibbidae + Bethylidae + Chrysididae	60	0.096774194
Plumariidae	20	Dryinidae + Embolemidae + Sclerogibbidae + Bethylidae + Chrysididae + Scolebythidae	63	0.487804878
Chrysidoidea	83	Vespoidea + Apoidea	197	0.594982079
Megalyridae	45	Trigonalidae	75	0.756302521
Stephanidae	100	Megalyridae + Trigonalidae	120	0.913242009
Megalyridae + Trigonalidae + Stephanidae	220	Aculeata	280	0.881763527
Aulacidae	150	Evaniidae	400	0.546448087
Gasteruptiidae	500	Aulacidae + Evaniidae	550	0.953288847
Ceraphronidae	360	Megaspilidae	450	0.889987639
Ceraphronoidea	810	Evanioidea	1050	0.871436256
Megalyridae + Trigonalidae + Stephanidae + Aculeata	500	Ceraphronoidea + Evanioidea	1860	0.423908436
Braconidae	40000	Ichneumonidae	60000	0.800008
Ceraphronoidea + Evanioidea + Megalyridae + Trigonalidae + Stephanidae + Aculeata	2360	Ichneumonoidea	100000	0.046112213
To be compared to Chalcidoidea + Proctotrupoidea + Cynipoidea + Mymarommatidae, but this clade needs correcting first				
Trichogrammatidae	675	Aphelinidae	1120	0.752508361
Mymarommatidae	9	Trichogrammatidae + Aphelinidae	1795	0.009983361
Mymarommatidae (A)	9	Signiphoridae (C)	80	0.204545455
Signiphoridae (C)	80	Trichogrammatidae + Aphelinidae (B)	1795	0.085378869
Signiphoridae	80	Mymarommatidae + Trichogrammatidae + Aphelinidae	1804	0.084970791
Mymaridae	1400	Signiphoridae + Mymarommatidae + Trichogrammatidae + Aphelinidae	1884	0.852878465
Agaonidae	650	Mymaridae + Signiphoridae + Mymarommatidae + Trichogrammatidae + Aphelinidae	3284	0.330536486
Ormyridae	90	Agaonidae + Mymaridae + Signiphoridae + Mymarommatidae + Trichogrammatidae + Aphelinidae	3934	0.044742729
Ormyridae (A)	90	Torymidae (C)	1150	0.14527845
Torymidae (C)	1150	Agaonidae + Mymaridae + Signiphoridae + Mymarommatidae + Trichogrammatidae + Aphelinidae (B)	3934	0.452488688
Torymidae	1150	Ormyridae + Agaonidae + Mymaridae + Signiphoridae + Mymarommatidae + Trichogrammatidae + Aphelinidae	4024	0.444616277
Eupelmidae	900	Encyrtidae	3825	0.381033023

Eupelmidae + Encyrtidae	4725	Torymidae + Ormyridae + Agaonidae + Mymaridae + Signiphoridae + Mymarommatidae + Trichogrammatidae + Aphelinidae	5174	0.954738331
Leucospidae	240	Chalcididae	1875	0.227057711
Eurytomidae	1425	Leucospidae + Chalcididae	2115	0.805312235
Eurytomidae + Leucospidae + Chalcididae	3540	Eupelmidae + Encyrtidae + Torymidae + Ormyridae + Agaonidae + Mymaridae + Signiphoridae + Mymarommatidae + Trichogrammatidae + Aphelinidae	9899	0.526864117
Pteromalidae	4115	Eurytomidae + Leucospidae + Chalcididae + Eupelmidae + Encyrtidae + Torymidae + Ormyridae + Agaonidae + Mymaridae + Signiphoridae + Mymarommatidae + Trichogrammatidae + Aphelinidae	13439	0.468865721
Elasmidae	260	Eulophidae	540	0.650813517
Tetracampidae	50	Elasmidae + Eulophidae	800	0.11778563
Perilampidae	260	Eucharitidae	380	0.813771518
Perilampidae + Eucharitidae	640	Elasmidae + Eulophidae + Tetracampidae	850	0.85963734
Perilampidae + Eucharitidae + Elasmidae + Eulophidae + Tetracampidae	1490	Pteromalidae + Eurytomidae + Leucospidae + Chalcididae + Eupelmidae + Encyrtidae + Torymidae + Ormyridae + Agaonidae + Mymaridae + Signiphoridae + Mymarommatidae + Trichogrammatidae + Aphelinidae	17554	0.156487948
Monomachidae	20	Diapriidae	2300	0.017248814
Monomachidae (A)	20	Perilampidae + Eucharitidae + Elasmidae + Eulophidae + Tetracampidae + Pteromalidae + Eurytomidae + Leucospidae + Chalcididae + Eupelmidae + Encyrtidae + Torymidae + Ormyridae + Agaonidae + Mymaridae + Signiphoridae + Mymarommatidae + Trichogrammatidae + Aphelinidae (C)	19044	0.002098306
Diapriidae (B)	2300	Perilampidae + Eucharitidae + Elasmidae + Eulophidae + Tetracampidae + Pteromalidae + Eurytomidae + Leucospidae + Chalcididae + Eupelmidae + Encyrtidae + Torymidae + Ormyridae + Agaonidae + Mymaridae + Signiphoridae + Mymarommatidae + Trichogrammatidae + Aphelinidae (C)	19044	0.215527339
Monomachidae + Diapriidae	4600	Perilampidae + Eucharitidae + Elasmidae + Eulophidae + Tetracampidae + Pteromalidae + Eurytomidae + Leucospidae + Chalcididae + Eupelmidae + Encyrtidae + Torymidae + Ormyridae + Agaonidae + Mymaridae + Signiphoridae + Mymarommatidae + Trichogrammatidae + Aphelinidae	19044	0.389121516
Vanhorniidae	5	Proctotrupidae	310	0.031847134
Vanhorniidae (A)	5	Heloridae (C)	7	0.909090909
Heloridae (C)	7	Proctotrupidae (B)	310	0.044303797
Heloridae	7	Vanhorniidae + Proctotrupidae	10	0.875
Proctotrupidae + Vanhorniidae + Heloridae	17	Monomachidae + Diapriidae + Perilampidae + Eucharitidae + Elasmidae + Eulophidae + Tetracampidae + Pteromalidae + Eurytomidae + Leucospidae + Chalcididae + Eupelmidae + Encyrtidae + Torymidae + Ormyridae + Agaonidae + Mymaridae + Signiphoridae + Mymarommatidae + Trichogrammatidae + Aphelinidae	23644	0.001437025

To be compared to Tanaostigmatidae + Platygasteroidea + Cynipoidea + Pelecinidae + Roproniidae, but this clade needs correcting first

Tanaostigmatidae	90	Scelionidae	3000	0.058271285
Platygasteridae	1100	Tanaostigmatidae + Scelionidae	3090	0.525185008
Ibaliidae	15	Cynipidae	2055	0.014499758
Ibaliidae (A)	15	Figitidae	250	0.113636364
Figitidae	250	Cynipidae (B)	2055	0.217013889
Figitidae	250	Ibaliidae + Cynipidae	2070	0.215610177
Liopteridae	71	Figitidae + Ibaliidae + Cynipidae	2320	0.059414226
Cynipoidea	2391	Tanaostigmatidae + Platygasteroidea	4190	0.72674772
Pelecinidae	1	Roproniidae	18	0.111111111
Pelecinidae + Roproniidae	19	Tanaostigmatidae + Platygasteroidea + Cynipoidea	6581	0.005758448
Proctotrupidae + Vanhorniidae + Heloridae	17	Monomachidae + Diapriidae + Perilampidae + Eucharitidae + Elasmidae + Eulophidae + Tetracampidae + Pteromalidae + Eurytomidae + Leucospidae + Chalcididae + Eupelmidae + Encyrtidae + Torymidae + Ormyridae + Agaonidae + Mymaridae + Signiphoridae + Mymarommatidae + Trichogrammatidae + Aphelinidae	23644	0.001437025

These two sister comaprisons are one another's outgroups. Since they cannot be corrected via comparison with an uncorrected outgroup they are combined into one larger clade. Previously a comparison of the two species poor lineages with the next outgroup and then the two species rich lineages with this outgroup is conducted. However the outgroup to these clades is Ichneumonoidea + Ceraphronoidea + Evanioidea + Stephanidae + Trigonalidae + Megalyridae + Aculeata, and is uncorrected itself

Ceraphronoidea + Evanioidea + Megalyridae + Trigonalidae + Stephanidae + Aculeata	2360	Ichneumonoidea	100000	0.046112213

Comparison to the next outgroup (Orussidae) is required

Orussidae (C)	70	Pelecinidae + Roproniidae & Proctotrupidae + Vanhorniidae + Heloridae & Ceraphronoidea + Evanioidea + Megalyridae + Trigonalidae + Stephanidae + Aculeata (A)	2396	0.056795132
Orussidae (C)	70	Tanaostigmatidae + Platygasteroidea + Cynipoidea & Monomachidae + Diapriidae + Perilampidae + Eucharitidae + Elasmidae + Eulophidae + Tetracampidae + Pteromalidae + Eurytomidae + Leucospidae + Chalcididae + Eupelmidae + Encyrtidae + Torymidae + Ormyridae + Agaonidae + Mymaridae + Signiphoridae + Mymarommatidae + Trichogrammatidae + Aphelinidae & Ichneumonoidea (B)	130225	0.001074493

This suggests that there has been a significant upshift in speciation in the lower branches of the Apocrita and that any significant differences at higher nodes represent downshifts?

Orussidae	70	Apocrita	4792	0.028800658
Orussidae (A)	70	Xiphydriidae (C)	80	0.939597315
Xiphydriidae (C)	80	Apocrita (B)	4792	0.032847465
Xiphydriidae	80	Apocrita + Orussidae	140	0.730593607
Anaxyelidae	1	Siricidae	93	0.021505376
Anaxyelidae (A)	1	Apocrita + Orussidae + Xiphydriidae (C)	220	0.009090909

Siricidae (B)	93	Apocrita + Orussidae + Xiphydriidae (C)	220	0.596153846
Siricoidea	186	Apocrita + Orussidae + Xiphydriidae	220	0.918518519
Cephidae	100	Apocrita + Orussidae + Xiphydriidae + Siricoidea	406	0.396039604
Megalodontidae	40	Pamphiliidae	200	0.334728033
Megalodontoidea	240	Apocrita + Orussidae + Xiphydriidae + Siricoidea + Cephidae	506	0.644295302
Pergidae	400	Argidae	870	0.630417652
Pterygophoridae	22	Argidae + Pergidae	1270	0.034082107
To be compared to Diprionidae + Cimbicidae + Tenthredinidae, but this clade needs correcting first				
Diprionidae	90	Cimbicidae	130	0.821917808
Diprionidae + Cimbicidae	220	Tenthredinidae	6000	0.070750925
Pterygophoridae (A)	22	Diprionidae + Cimbicidae + Tenthredinidae (C)	6220	0.007050152
Argidae + Pergidae (B)	1270	Diprionidae + Cimbicidae + Tenthredinidae (C)	6220	0.339164107
Argidae + Pergidae + Pterygophoridae	2540	Diprionidae + Cimbicidae + Tenthredinidae	6220	0.579974883
Blasticotomidae	9	Diprionidae + Cimbicidae + Tenthredinidae + Argidae + Pergidae + Pterygophoridae	8760	0.00205292
Blasticotomidae (A)	9	Apocrita + Orussidae + Xiphydriidae + Siricoidea + Cephidae + Megalodontoidea (C)	746	0.023872679
Apocrita + Orussidae + Xiphydriidae + Siricoidea + Cephidae + Megalodontoidea (C)	746	Diprionidae + Cimbicidae + Tenthredinidae + Argidae + Pergidae + Pterygophoridae (B)	8760	0.156970016
Apocrita + Orussidae + Xiphydriidae + Siricoidea + Cephidae + Megalodontoidea	746	Tenthredinoidea (B)	17520	0.081686285
Xyelidae	46	Apocrita + Orussidae + Xiphydriidae + Siricoidea + Cephidae + Megalodontoidea + Diprionidae + Cimbicidae + Tenthredinidae + Argidae + Pergidae + Pterygophoridae + Blasticotomidae	18266	0.005024302
Xyelidae (A)	46	Panorpida (C)	284650	0.000323153
Apocrita + Orussidae + Xiphydriidae + Siricoidea + Cephidae + Megalodontoidea + Diprionidae + Cimbicidae + Tenthredinidae + Argidae + Pergidae + Pterygophoridae + Blasticotomidae (B)	18266	Panorpida (C)	284650	0.120601489

MRC Compartmentalised

Smaller Taxon	Species	Larger taxon	Species	p (two-tailed)
Megachilidae	3000	Anthophoridae	4000	0.857265324
Apidae	1000	Anthophoridae + Megachilidae	7000	0.250031254
Melittidae	100	Anthophoridae + Apidae + Megachilidae	8000	0.024694407
Oxaeidae	20	Adrenidae	2000	0.019811788
These two sister comaprisons are one another's outgroups. Since they cannot be corrected via comparison with an uncorrected outgroup they are combined into one larger clade. A comparison of the two species poor lineages with the next outgroup (Halictidae) and then the two species rich lineages with this outgroup is conducted.				
Melittidae & Oxaeidae (A)	120	Halictidae (C)	3500	0.066316662
Halictidae (C)	3500	Adrenidae & Anthophoridae + Apidae + Megachilidae (B)	10000	0.51855693

Halictidae	3500	Oxaeidae + Adrenidae + Anthophoridae + Apidae + Megachilidae + Melittidae	10120	0.513987811
Stenotritidae	30	Colletidae	2000	0.029571217
Stenotritidae (A)	30	Halictidae + Oxaeidae + Adrenidae + Anthophoridae + Apidae + Megachilidae + Melittidae (C)	13620	0.004395926
Colletidae (B)	2000	Halictidae + Oxaeidae + Adrenidae + Anthophoridae + Apidae + Megachilidae + Melittidae (C)	13620	0.256098342
Stenotritidae + Colletidae	4000	Halictidae + Oxaeidae + Adrenidae + Anthophoridae + Apidae + Megachilidae + Melittidae (C)	13620	0.454055281
Sphecidae	6020	Anthophila	17620	0.072214016
Heterogynidae	5	Anthophila + Sphecidae	23640	0.000546926
Heterogynidae (A)	5	Ampulicidae (C)	167	0.058479532
Ampulicidae (C)	167	Anthophila + Sphecidae (B)	23640	0.018106907
Anthophila + Sphecidae + Heterogynidae	10	Ampulicidae	167	0.113636364
Scoliidae	300	Vespidae	4000	0.139567341
Scoliidae + Vespidae	4300	Formicidae	8800	0.656538667
Bradynobaenidae	155	Scoliidae + Vespidae + Formicidae	13100	0.023389166
To be compared to Rhopalosomatidae + Pompillidae, but this clade needs correcting first				
Rhopalosomatidae	34	Pompillidae	4200	0.016064257
These two sister comaprisons are one another's outgroups. Since they cannot be corrected via comparison with an uncorrected outgroup they are combined into one larger clade. A comparison of the two species poor lineages with the next outgroup and then the two species rich lineages with this outgroup is conducted. This outgroup is Tiphiidae + Mutillidae + Sapygidae and needs correcting first.				
Sapygidae	80	Mutillidae	5000	0.031502264
Sapygidae (A)	80	Tiphiidae (C)	1500	0.101329956
Tiphiidae (C)	1500	Mutillidae (B)	5000	0.461609478
Tiphiidae	1500	Sapygidae + Mutillidae	5080	0.455996352
Bradynobaenidae & Rhopalosomatidae (A)	189	Sapygidae + Mutillidae + Tiphiidae (C)	6580	0.055851064
Sapygidae + Mutillidae + Tiphiidae (C)	6580	Scoliidae + Vespidae + Formicidae & Pompillidae (B)	17300	0.551111856
Sapygidae + Mutillidae + Tiphiidae	6580	Bradynobaenidae + Scoliidae + Vespidae + Formicidae + Rhopalosomatidae + Pomillidae	17489	0.546784112
Apoidea	177	Sapygidae + Mutillidae + Tiphiidae + Bradynobaenidae + Scoliidae + Vespidae + Formicidae + Rhopalosomatidae + Pomillidae	24069	0.014600949
Sierolomorphidae (C)	10	Apoidea (corr. Anthophila + Sphecidae, Steno) (A)	177	0.107526882
Sierolomorphidae (C)	10	Sapygidae + Mutillidae + Tiphiidae + Bradynobaenidae + Scoliidae + Vespidae + Formicidae + Rhopalosomatidae + Pomillidae (B)	24069	0.000830634
Sierolomorphidae	10	(Sapygidae + Mutillidae + Tiphiidae + Bradynobaenidae + Scoliidae + Vespidae + Formicidae + Rhopalosomatidae + Pomillidae) + Apoidea (corr. Anthophila + Sphecidae, Steno)	354	0.055096419
Embolemidae	10	Dryinidae	1100	0.018034265
Embolemidae (A)	10	Sclerogibbidae (C)	10	1.052631579
Sclerogibbidae (C)	10	Dryinidae (B)	1100	0.018034265
Sclerogibbidae	10	Dryinidae + Embolemidae	20	0.689655172

Bethylidae	2200	Chrysididae	3000	0.846316599
Dryinidae + Embolemidae + Sclerogibbidae	30	Bethylidae + Chrysididae	5200	0.011474469
Scolebythidae (C)	3	Dryinidae + Embolemidae + Sclerogibbidae (A)	30	0.1875
Scolebythidae (C)	3	Bethylidae + Chrysididae (B)	5200	0.001153403
Scolebythidae	3	Dryinidae + Embolemidae + Sclerogibbidae + Bethylidae + Chrysididae	60	0.096774194
Plumariidae	20	Dryinidae + Embolemidae + Sclerogibbidae + Bethylidae + Chrysididae + Scolebythidae	63	0.487804878
Chrysidoidea	83	Apoidea + Vespoidea	364	0.372197309
Stephanidae	100	Aculeata	447	0.366300366
Trichogrammatidae	675	Aphelinidae	1120	0.752508361
Mymarommatidae	9	Trichogrammatidae + Aphelinidae	1795	0.009983361
Mymarommatidae (A)	9	Signiphoridae (C)	80	0.204545455
Signiphoridae (C)	80	Trichogrammatidae + Aphelinidae (B)	1795	0.085378869
Signiphoridae	80	Mymarommatidae + Trichogrammatidae + Aphelinidae	1804	0.084970791
Mymaridae	1400	Signiphoridae + Mymarommatidae + Trichogrammatidae + Aphelinidae	1884	0.852878465
Agaonidae	650	Mymaridae + Signiphoridae + Mymarommatidae + Trichogrammatidae + Aphelinidae	3284	0.330536486
Ormyridae	90	Agaonidae + Mymaridae + Signiphoridae + Mymarommatidae + Trichogrammatidae + Aphelinidae	3934	0.044742729
Ormyridae (A)	90	Torymidae (C)	1150	0.14527845
Torymidae (C)	1150	Agaonidae + Mymaridae + Signiphoridae + Mymarommatidae + Trichogrammatidae + Aphelinidae (B)	3934	0.452488688
Torymidae	1150	Ormyridae + Agaonidae + Mymaridae + Signiphoridae + Mymarommatidae + Trichogrammatidae + Aphelinidae	4024	0.444616277
Eupelmidae	900	Encyrtidae	3825	0.381033023
Eupelmidae + Encyrtidae	4725	Torymidae + Ormyridae + Agaonidae + Mymaridae + Signiphoridae + Mymarommatidae + Trichogrammatidae + Aphelinidae	5174	0.954738331
Chalcididae	1875	Eupelmidae + Encyrtidae + Torymidae + Ormyridae + Agaonidae + Mymaridae + Signiphoridae + Mymarommatidae + Trichogrammatidae + Aphelinidae	9899	0.31852544
Eulophidae	540	Pteromalidae	4115	0.232058444
Elasmidae	260	Pteromalidae + Eulophidae	4655	0.105820106
Tetracampidae	50	Pteromalidae + Eulophidae + Elasmidae	4915	0.020145044
To be compared to Perilampidae + Eucharitidae, but this clade needs correcting first				
Perilampidae	260	Eucharitidae	380	0.813771518
Tetracampidae (A)	50	Perilampidae + Eucharitidae (C)	640	0.145137881
Perilampidae + Eucharitidae (C)	640	Pteromalidae + Eulophidae + Elasmidae (B)	4915	0.23046453
Perilampidae + Eucharitidae	640	Pteromalidae + Eulophidae + Elasmidae + Tetracampidae	4965	0.22840828

Tanaostigmatidae	90	Chalcididae + Eupelmidae + Encyrtidae + Torymidae + Ormyridae + Agaonidae + Mymaridae + Signiphoridae + Mymarommatidae + Pteromalidae + Eulophidae + Elasmidae + Tetracampidae + Perilampidae + Eucharitidae + Leucospidae	17619	0.010164897

To be compared to Monomachidae + Diapriidae, but this clade needs correcting first

Monomachidae	20	Diapriidae	2300	0.017248814

These two sister comaprisons are one another's outgroups. Since they cannot be corrected via comparison with an uncorrected outgroup they are combined into one larger clade. A comparison of the two species poor lineages with the next outgroup and then the two species rich lineages with this outgroup is conducted. This outgroup is Heloridae + Proctotrupidae + Vanhorniidae and needs correcting first.

Vanhorniidae	5	Proctotrupidae	310	0.031847134
Vanhorniidae (A)	5	Heloridae (C)	7	0.909090909
Heloridae (C)	7	Proctotrupidae (B)	310	0.044303797
Heloridae	7	Vanhorniidae + Proctotrupidae	10	0.875
Vanhorniidae + Proctotrupidae + Heloridae (C)	17	Tanaostigmatidae & Monomachidae (A)	110	0.26984127
Vanhorniidae + Proctotrupidae + Heloridae (C)	17	Chalcididae + Eupelmidae + Encyrtidae + Torymidae + Ormyridae + Agaonidae + Mymaridae + Signiphoridae + Mymarommatidae + Pteromalidae + Eulophidae + Elasmidae + Tetracampidae + Perilampidae + Eucharitidae + Leucospidae & Diapriidae (B)	19919	0.001705543
Vanhorniidae + Proctotrupidae + Heloridae	17	Chalcididae + Eupelmidae + Encyrtidae + Torymidae + Ormyridae + Agaonidae + Mymaridae + Signiphoridae + Mymarommatidae + Pteromalidae + Eulophidae + Elasmidae + Tetracampidae + Perilampidae + Eucharitidae + Leucospidae + Tanaostigmatidae + Monomachidae + Diapriidae	220	0.144067797

Ibaliidae	15	Cynipidae	2055	0.014499758
Ibaliidae (A)	15	Figitidae (C)	250	0.113636364
Figitidae (C)	250	Cynipidae (B)	2055	0.217013889
Figitidae	250	Ibaliidae + Cynipidae	2070	0.215610177
Liopteridae	71	Figitidae + Ibaliidae + Cynipidae	2320	0.059414226
Platygasteridae	1100	Scelionidae	3000	0.536716272
Cynipoidea	2391	Platygasteridae + Scelionidae	4100	0.736825886
Pelecinidae	1	Roproniidae	18	0.111111111
Pelecinidae + Roproniidae	19	Cynipoidea + Platygasteridae + Scelionidae	6491	0.00583807
Pelecinidae + Roproniidae (A)	19	Vanhorniidae + Proctotrupidae + Heloridae + Chalcididae + Eupelmidae + Encyrtidae + Torymidae + Ormyridae + Agaonidae + Mymaridae + Signiphoridae + Mymarommatidae + Pteromalidae + Eulophidae + Elasmidae + Tetracampidae + Perilampidae + Eucharitidae + Leucospidae + Tanaostigmatidae + Monoachidae + Diapriidae (C)	237	0.149019608
Vanhorniidae + Proctotrupidae + Heloridae + Chalcididae + Eupelmidae + Encyrtidae + Torymidae + Ormyridae + Agaonidae + Mymaridae + Signiphoridae + Mymarommatidae + Pteromalidae + Eulophidae + Elasmidae + Tetracampidae + Perilampidae + Eucharitidae + Leucospidae + Tanaostigmatidae + Monomachidae + Diapriidae (C)	237	Cynipoidea + Platygasteridae + Scelionidae (B)	6491	0.070462316

Braconidae	40000	Ichneumonidae	60000	0.800008
Cynipoidea + Platygasteridae + Scelionidae + Proctotrupoidea + Chalcidoidea	6510	Ichneumonoidea	100000	0.122243191
Trigonalidae	75	Ichneumonoidea + Cynipoidea + Platygasteridae + Scelionidae + Proctotrupoidea + Chalcidoidea	106510	0.001407341
Trigonalidae (A)	75	Aculeata + Stephanidae (C)	547	0.241545894
Aculeata + Stephanidae (C)	547	Ichneumonoidea + Cynipoidea + Platygasteridae + Scelionidae + Proctotrupoidea + Chalcidoidea (B)	106510	0.010218951
Trigonalidae + Ichneumonoidea + Cynipoidea + Platygasteridae + Scelionidae + Proctotrupoidea + Chalcidoidea	150	Aculeata + Stephanidae	547	0.431034483
Aulacidae	150	Gasteruptiidae	500	0.462249615
Evaniidae	400	Aulacidae + Gasteruptiidae	650	0.762631077
Ceraphronidae	360	Megaspilidae	450	0.889987639
Ceraphronoidea	810	Evanioidea	1050	0.871436256
Aculeata + Stephanidae + Trigonalidae + Ichneumonoidea + Cynipoidea + Platygasteridae + Scelionidae + Proctotrupoidea + Chalcidoidea	697	Ceraphronoidea + Evanioidea	1860	0.545383412
Megalyridae	45	Eurytomidae	1425	0.061266167
Megalyridae + Eurytomidae	1470	Ceraphronoidea + Evanioidea + Aculeata + Stephanidae + Trigonalidae + Ichneumonoidea + Cynipoidea + Platygasteridae + Scelionidae + Proctotrupoidea + Chalcidoidea	2557	0.730253353
Orussidae	70	Apocrita	4027	0.034179688
Orussidae (A)	70	Xiphydriidae (C)	80	0.939597315
Xiphydriidae (C)	80	Apocrita (B)	4027	0.038967365
Xiphydriidae	80	Apocrita + Orussidae	140	0.730593607
Anaxyelidae	1	Siricidae	93	0.021505376
Anaxyelidae (A)	1	Apocrita + Orussidae + Xiphydriidae (C)	220	0.009090909
Siricidae (B)	93	Apocrita + Orussidae + Xiphydriidae (C)	220	0.596153846
Siricoidea	186	Apocrita + Orussidae + Xiphydriidae	220	0.918518519
Cephidae	100	Apocrita + Orussidae + Xiphydriidae + Siricoidea	406	0.396039604
Megalodontidae	40	Pamphiliidae	200	0.334728033
Megalodontoidea	240	Apocrita + Orussidae + Xiphydriidae + Siricoidea + Cephidae	506	0.644295302
Cimbicidae	130	Tenthredinidae	6000	0.042421276
Diprionidae (C)	90	Cimbicidae (A)	130	0.821917808
Diprionidae (C)	90	Tenthredinidae (B)	6000	0.029561504
Diprionidae	90	Cimbicidae + Tenthredinidae	260	0.515759312
Pergidae	400	Argidae	870	0.630417652
Pterygophoridae	22	Argidae + Pergidae	1270	0.034082107
Pterygophoridae (A)	22	Diprionidae + Cimbicidae + Tenthredinidae (C)	350	0.118598383
Diprionidae + Cimbicidae + Tenthredinidae (C)	350	Argidae + Pergidae (B)	1270	0.432365658
Diprionidae + Cimbicidae + Tenthredinidae	350	Pterygophoridae + Argidae + Pergidae	1292	0.426569165
Blasticotomidae	9	Diprionidae + Cimbicidae + Tenthredinidae + Pterygophoridae + Argidae + Pergidae	1642	0.010909091

Blasticotomidae (A)	9	Apocrita + Orussidae + Xiphydriidae + Siricoidea + Cephidae + Megalodontoidea (C)	746	0.023872679
Apocrita + Orussidae + Xiphydriidae + Siricoidea + Cephidae + Megalodontoidea (C)	746	Diprionidae + Cimbicidae + Tenthredinidae + Pterygophoridae + Argidae + Pergidae (B)	1642	0.625052367
Apocrita + Orussidae + Xiphydriidae + Siricoidea + Cephidae + Megalodontoidea	746	Tenthredinoidea	3284	0.370315215
Xyelidae	46	Apocrita + Orussidae + Xiphydriidae + Siricoidea + Cephidae + Megalodontoidea + Tenthredinoidea	4030	0.022576687
Xyelidae (A)	46	Panorpida (C)	284650	0.000323153
Apocrita + Orussidae + Xiphydriidae + Siricoidea + Cephidae + Megalodontoidea + Tenthredinoidea (B)	4030	Panorpida (C)	284650	0.027920285

Standard MRP

This analysis was conducted to see if the alternative internal phylogeny for Vespoidea would affect the detection of a shift at the origin of the Vespoidea.

Smaller Taxon	Species	Larger taxon	Species	p (two-tailed)
Megachilidae	3000	Anthophoridae	4000	0.857265324
Apidae	1000	Anthophoridae + Megachilidae	7000	0.250031254
Melittidae	100	Anthophoridae + Apidae + Megachilidae	8000	0.024694407
Oxaeidae	20	Adrenidae	2000	0.019811788
These two sister comaprisons are one another's outgroups. Since they cannot be corrected via comparison with an uncorrected outgroup they are combined into one larger clade. A comparison of the two species poor lineages with the next outgroup (Halictidae) and then the two species rich lineages with this outgroup is conducted.				
Melittidae & Oxaeidae (A)	120	Halictidae (C)	3500	0.066316662
Halictidae (C)	3500	Adrenidae & Anthophoridae + Apidae + Megachilidae (B)	10000	0.51855693
Halictidae	3500	Oxaeidae + Adrenidae + Anthophoridae + Apidae + Megachilidae + Melittidae	10120	0.513987811
Stenotritidae	30	Colletidae	2000	0.029571217
Stenotritidae (A)	30	Halictidae + Oxaeidae + Adrenidae + Anthophoridae + Apidae + Megachilidae + Melittidae (C)	13620	0.004395926
Colletidae (B)	2000	Halictidae + Oxaeidae + Adrenidae + Anthophoridae + Apidae + Megachilidae + Melittidae (C)	13620	0.256098342
Stenotritidae + Colletidae	4000	Halictidae + Oxaeidae + Adrenidae + Anthophoridae + Apidae + Megachilidae + Melittidae (C)	13620	0.454055281
Sphecidae	660	Anthophila	17620	0.072214016
Heterogynidae	5	Anthophila + Sphecidae	18280	0.000546926
Heterogynidae (A)	5	Ampulicidae (C)	167	0.058479532
Ampulicidae (C)	167	Anthophila + Sphecidae (B)	18280	0.018106907
Anthophila + Sphecidae + Heterogynidae	10	Ampulicidae	167	0.113636364

Scoliidae	300	Vespidae	4000	0.139567341
Bradynobaenidae	155	Scoliidae + Vespidae	4300	0.069600359
Sapygidae	80	Mutillidae	5000	0.031502264
Sapygidae (A)	80	Tiphiidae (C)	1500	0.101329956
Tiphiidae (C)	1500	Mutillidae (B)	5000	0.461609478
Tiphiidae	1500	Sapygidae + Mutillidae	5080	0.455996352
Scoliidae + Vespidae + Bradynobaenidae	4455	Sapygidae + Mutillidae + Tiphiidae	6580	0.807504078
Sierolomorphidae	10	Formicidae	8800	0.002270405
Sierolomorphidae (A)	10	Sapygidae + Mutillidae + Tiphiidae + Scoliidae + Vespidae + Bradynobaenidae (C)	11035	0.001810938
Formicidae (B)	8800	Sapygidae + Mutillidae + Tiphiidae + Scoliidae + Vespidae + Bradynobaenidae (C)	11035	0.887365131
Sapygidae + Mutillidae + Tiphiidae + Scoliidae + Vespidae + Bradynobaenidae	11035	Sierolomophidae + Formicidae	17600	0.770762031
Rhopalosomatidae	34	Pompillidae	4200	0.016064257
Rhopalosomatidae (A)	34	Sapygidae + Mutillidae + Tiphiidae + Scoliidae + Vespidae + Bradynobaenidae + Sierolomophidae + Formicidae (C)	28635	0.002371983
Pompillidae (B)	4200	Sapygidae + Mutillidae + Tiphiidae + Scoliidae + Vespidae + Bradynobaenidae + Sierolomophidae + Formicidae (C)	28635	0.255832369
Rhopalosomatidae + Pompillidae	8400	Sapygidae + Mutillidae + Tiphiidae + Scoliidae + Vespidae + Bradynobaenidae + Sierolomophidae + Formicidae	28635	0.453637198
Apoidea	177	Vespoidea	28635	0.012286974
Embolemidae	10	Dryinidae	1100	0.018034265
Embolemidae (A)	10	Sclerogibbidae (C)	10	1.052631579
Sclerogibbidae (C)	10	Dryinidae (B)	1100	0.018034265
Sclerogibbidae	10	Dryinidae + Embolemidae	20	0.689655172
Bethylidae	2200	Chrysididae	3000	0.846316599
Dryinidae + Embolemidae + Sclerogibbidae	30	Bethylidae + Chrysididae	5200	0.011474469
Scolebythidae (C)	3	Dryinidae + Embolemidae + Sclerogibbidae (A)	30	0.1875
Scolebythidae (C)	3	Bethylidae + Chrysididae (B)	5200	0.001153403
Scolebythidae	3	Dryinidae + Embolemidae + Sclerogibbidae + Bethylidae + Chrysididae	60	0.096774194
Plumariidae	20	Dryinidae + Embolemidae + Sclerogibbidae + Bethylidae + Chrysididae + Scolebythidae	63	0.487804878
Chrysidoidea (C)	83	Apoidea (A)	177	0.640926641
Chrysidoidea (C)	84	Vespoidea (B)	28635	0.00584999
